# Senescent Fibroblasts Enhance Early Skin Carcinogenic Events via a Paracrine MMP-PAR-1 Axis

**DOI:** 10.1371/journal.pone.0063607

**Published:** 2013-05-10

**Authors:** Nicolas Malaquin, Chantal Vercamer, Fatima Bouali, Sébastien Martien, Emeric Deruy, Nicolas Wernert, Maggy Chwastyniak, Florence Pinet, Corinne Abbadie, Albin Pourtier

**Affiliations:** 1 Unité Mixte de Recherche UMR 8161, Centre National de la Recherche Scientifique, CNRS, Institut de Biologie de Lille, Lille, France; 2 Université Lille1 Sciences et Technologies, UMR 8161, Villeneuve d’Ascq, France; 3 Université Lille2 Droit et Santé, UMR 8161, Lille, France; 4 Institut Pasteur de Lille, Lille, France; 5 Institut National de la Santé et de la Recherche Médicale INSERM, Unité Mixte de Recherche UMR 744, Lille, France; 6 Molecular Pathology Department, Institute of Pathology, University of Bonn, Bonn, Germany; University of Maryland School of Medicine, United States of America

## Abstract

The incidence of carcinoma increases greatly with aging, but the cellular and molecular mechanisms underlying this correlation are only partly known. It is established that senescent fibroblasts promote the malignant progression of already-transformed cells through secretion of inflammatory mediators. We investigated here whether the senescent fibroblast secretome might have an impact on the very first stages of carcinogenesis. We chose the cultured normal primary human epidermal keratinocyte model, because after these cells reach the senescence plateau, cells with transformed and tumorigenic properties systematically and spontaneously emerge from the plateau. In the presence of medium conditioned by autologous senescent dermal fibroblasts, a higher frequency of post-senescence emergence was observed and the post-senescence emergent cells showed enhanced migratory properties and a more marked epithelial-mesenchymal transition. Using pharmacological inhibitors, siRNAs, and blocking antibodies, we demonstrated that the MMP-1 and MMP-2 matrix metalloproteinases, known to participate in late stages of cancer invasion and metastasis, are responsible for this enhancement of early migratory capacity. We present evidence that MMPs act by activating the protease-activated receptor 1 (PAR-1), whose expression is specifically increased in post-senescence emergent keratinocytes. The physiopathological relevance of these results was tested by analyzing MMP activity and PAR-1 expression in skin sections. Both were higher in skin sections from aged subjects than in ones from young subjects. Altogether, our results suggest that during aging, the dermal and epidermal skin compartments might be activated coordinately for initiation of skin carcinoma, via a paracrine axis in which MMPs secreted by senescent fibroblasts promote very early epithelial-mesenchymal transition of keratinocytes undergoing transformation and oversynthesizing the MMP-activatable receptor PAR-1.

## Introduction

Carcinomas are by far the most frequent cancers in humans. While their incidence is almost zero before the age of 20, it reaches a peak between ages 45 and 75, depending on the type of carcinoma (NCI and WHO data). The molecular and cellular mechanisms underlying this relationship between advanced age and carcinogenesis remain unclear. During aging, senescent cells accumulate in both the epithelial and stromal tissues of healthy organs [1], [2]. They are also found in precancerous and cancerous lesions, again in both tumoral epithelial and non-tumoral stromal tissues [3], [4], [5], [6], [7]. Senescence is assumed to be a cell-autonomous tumor-suppressor mechanism, because it is accompanied by irreversible cell-cycle arrest occurring mainly in response to irreparable telomeric and non-telomeric DNA damage [8], [9]. This has been especially well demonstrated for fibroblasts, the major cell component of the stroma. Yet fibroblast senescence may contribute to promoting cancer development and evolution, in a non-cell-autonomous, paracrine way, as suggested by the observation that senescent fibroblasts can stimulate growth, the epithelial-mesenchymal transition (EMT), and invasiveness of premalignant and malignant cells [7], [10], [11], [12]. This results from the fact that senescing fibroblasts develop a senescence-associated secretory phenotype (SASP) similar to that of carcinoma-associated fibroblasts, characterized by increased expression and secretion of growth factors, inflammatory cytokines, and matrix metalloproteinases [10], [13], [14], [15]. These findings, however, do not directly explain why the incidence of carcinoma increases with age. Since the SASP has no effect on normal epithelial cells [11], specific molecular changes are expected to occur in aging epithelial cells, sensitizing them to the SASP promotion of carcinoma development.

The cell-autonomous tumor-suppressive character of senescence is less clear for several types of epithelial cells and melanocytes than for fibroblasts. Almost all precancerous cells of benign tumors display senescence markers, which are lost in the subsequent malignant tumors [3], [4], [5], [6]. This suggests that in epithelial cells and melanocytes, senescence is only a transitory barrier that is overcome in a significant number of cases. Senescence evasion can be achieved through alteration of the functions of major tumor suppressor genes, such as p16INK4, whose inactivation allows S-phase re-entry [16], and oncogenes such as TWIST and Ras, whose co-activation leads to a strong EMT [17].

Non-melanoma skin carcinomas (NMSCs) are the commonest cancers in the aging populations of developed countries, and their incidence is on the increase in association with rising life expectancy. More than 2 million cases of NMSCs were estimated in 2010 in the United States [18]. Because of their high frequency, NMSCs, especially squamous cell carcinomas that can evolve as metastatic, cause considerable morbidity and greater mortality than Hodgkin's lymphoma or thyroid, bone, or testicle cancer [19]. Interestingly, the occurrence of an NMSC is associated with an increased risk of developing a second primary carcinoma [20]. Therefore, the study of NMSCs may shed light on general features of initial mechanisms of carcinogenesis associated with aging. A common hypothesis is that the increase in carcinoma incidence with age might result from changes in the aging stroma.

When put in primary culture, the two major skin cell types, normal human dermal fibroblasts (NHDFs) and normal human epidermal keratinocytes (NHEKs), behave differently regarding senescence and senescence evasion, in a way that seems relevant to the *in vivo* and epidemiological findings described above. Like other fibroblasts [21], [22], NHDFs, after 50–60 population doublings (PDs), enter a senescence plateau which is irreversible and associated with shortened telomeres (Gosselin, Martien, Deruy and Abbadie, unpublished data). NHEKs, on the other hand, enter senescence after only 10–20 PDs, because of oxidative stress resulting at least partly from activation of the NF-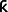
B/MnSOD/H_2_O_2_ pathway [23], and at this time they still display long telomeres. While accumulated oxidative damage results in death by autophagy of most senescent NHEKs [24], [25], a fraction of the senescent population (10^−4^ to 10^−2^) systematically and spontaneously re-enters in the cell cycle, generating clones of daughter cells that resume growth [26]. These post-senescence emergent keratinocytes (PSE-NHEKs) display a modified transcriptome, reflecting a certain degree of transformation, and a slight decline in E-cadherin expression, indicating that they have undergone a slight EMT. Remarkably, despite this apparently moderate transformed *in vitro* phenotype, PSE-NHEKs form, within 8 months after being xenografted into flanks of nude mice, disseminated skin hyperplasias and small carcinomas [26], indicative of initial intrinsic *in vitro* molecular changes allowing neoplastic development. Thus, the *in vitro* post-senescence neoplastic emergence (PSNE) of NHEKs might involve the early molecular events enabling the generation of transformed epithelial cells in aged individuals.

Here we have investigated whether and how the secretome of senescent NHDFs might favor PSNE or modify the properties of PSE-NHEKs. We show that NHEKs exposed throughout their culture to factors secreted by senescent NHDFs undergo PSNE more frequently than unexposed cells, and that EMT is enhanced in the resulting PSE-NHEKs. We further show that the active MMP-1 and MMP-2 matrix metalloproteinases, present in the secretome of senescent NHDFs, can trigger PSE-NHEK migration by activating the protease-activated receptor-1 (PAR-1), whose synthesis is remarkably upregulated in PSE-NHEKs. Our results indicate that the intrinsic transforming molecular changes enabling NHEKs to evade senescence are enhanced by the senescing stromal microenvironment and include acquisition of the ability to respond to the pro-EMT and pro-migratory influence of this microenvironment. They suggest that additive and cooperative cell-autonomous and paracrine mechanisms occurring during senescence may contribute to the earliest stages of carcinogenesis, including early EMT and early dissemination.

## Materials and Methods

### Cell Culture, Preparation, and use of Conditioned Media, Calculation of PSNE Frequency

NHEKs and NHDFs used in this study were NHEKs 2F1958 (Cambrex CC-2501) and NHDFs 2F1966 (Cambrex CC-2511) from a 37-year-old Caucasian female, NHEKs 1619 and NHDFs 1619 (Tebu-bio 102-05a) from a 19-year-old Caucasian female. Other NHEKs were used alone in some complementary experiments: NHEKs 1F2255, from a 40-year-old Asian female (Cambrex CC-2501) and NHEKs 4F0315, from a 31-year-old Caucasian female (Cambrex CC-2501).

NHDFs and NHEKs were cultured as described in [26], [27]. Cells were plated at 200,000 cells per 100-mm dish and cultures were always split at 70% confluence. The number of population doublings (PDs) was calculated as follows: *PD = ln(number of collected cells/number of plated cells)/ln2.*

NHDFs were grown in FGM-2 BulletKit medium® (CC-4126, Lonza), here called FGM. “Young NHDFs” refers to NHDFs dividing regularly during the exponential growth phase below 20 PDs. “Senescent NHDFs” refers to NHDFs in cultures having reached the senescence plateau. As described previously [27], the latter display the typical hallmarks of senescence: growth arrest, enlarged and flattened morphology, an increased number of vesicles, and Senescence-Associated beta-Galactosidase (SA-β-Gal) activity [1] (Figure S1). Up to now we performed cultures of NHDFs from four different donors. Once senescent, NHDFs can be kept in culture for up to two years by simply renewing the culture medium without displaying significant cell death or resuming growth (unpublished data).

**Figure 1:**
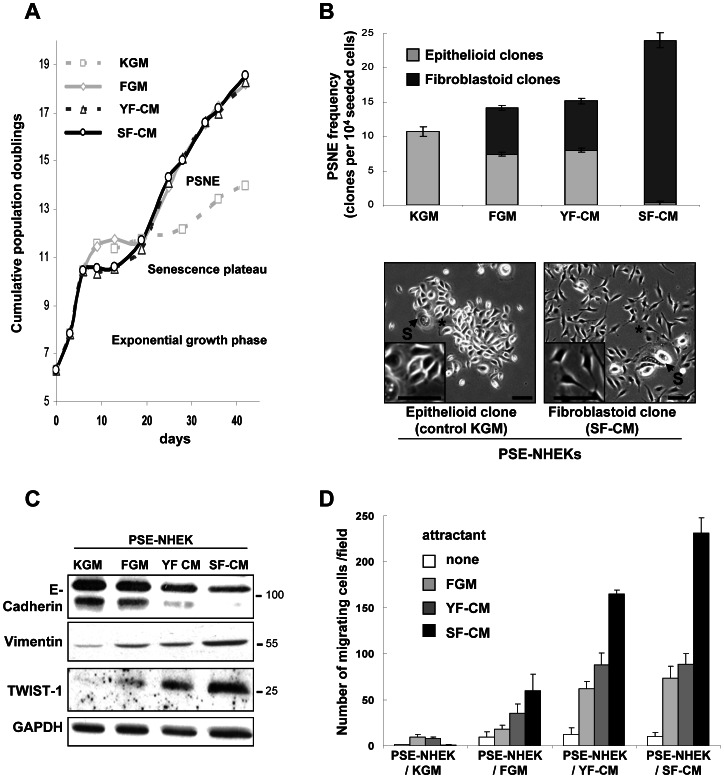
Impact of senescent-fibroblast-conditioned medium on keratinocyte growth, senescence, post-senescence emergence and the epithelial-mesenchymal transition. **A.** Growth curves of NHEKs (donor 2F1958) cultured for 45 days in either control keratinocyte medium (KGM), 90% KGM+10% fresh fibroblast growth medium (FGM), 90% KGM+10% young fibroblast-conditioned medium (YF-CM), or 90% KGM+10% senescent fibroblast-conditioned medium (SF-CM). (PSNE =  Post Senescence Neoplastic Emergence). The graph is representative of eight independent experiments. **B.** Senescent NHEKs cultured under the four above-described conditions were plated at low density (10,000 cells per 100 mm dish). After a week, the total number of epithelioid (gray) and fibroblastoid (black) post-senescence clones was counted in each culture dish (n = 6). The PSNE frequency was calculated as described under Materials and Methods. The graph shows the mean PSNE frequency±SD. Results of statistical analyses are given in Figure S2A. Results are representative of two independent experiments performed with cells from donor 2F1958. Representative images of epithelioid PSE-NHEKs clones from control culture conditions, and fibroblastoid PSE-NHEKs clones from co-culture with SF-CM are shown. Senescent cells (S) amongst PSE cells are indicated. Inserts show magnification of areas marked by an asterisk. Bar  = 100 µm. Two duplicated independent experiments with NHEKs and conditioned media from autologous NHDFs from two other donors showed a similar promotion of fibroblastoid PSE-NHEK emergence. **C.** Western blot analyses showing levels of molecular EMT markers (E-cadherin, vimentin, and TWIST-1) in PSE-NHEKs obtained under the four above-mentioned culture conditions. Quantitative analysis is provided in Figure S2C. Results are representative of two complete cultures and of independent western blotting experiments performed with material from donor 2F1958. D. Migration assays. PSE-NHEKs cultured as described above were starved in fresh KBM for 16 h, trypsinized, and seeded in KBM into Transwell® plates. FGM, YF-CM, or SF-CM was placed in the lower chamber as attractant. After 30 h of incubation at 37°C, cells having migrated were counted (ten random fields per well were counted, and each condition was in triplicate). Results are given as mean ± SD. Similar results were obtained in three independent experiments with cells from donor 2F1958. Statistical analyses are detailed in Figure S2D.

NHEKs were grown at 37°C in KGM-2 BulletKit medium® (CC-4152 Lonza), here called KGM, in an atmosphere containing 5% CO_2_. “Young NHEKs” refers to NHEKs dividing regularly during the exponential growth phase and growing in islets, whereas “senescent NHEKs” refers to NHEKs in cultures having reached the senescence growth plateau. The latter also display the typical hallmarks of senescence, as previously published [23], [24], [25], [26] (Figure 1). “PSE-NHEKs” refers to the population emerging from the senescence plateau. These cells have been characterized in a previous study: they grow in islets, have an epithelioid morphology, divide actively, and have lost the SA-β-Gal marker. They rapidly replace the senescent cells and fill the dish (Figure 1). So far, we have recorded the occurrence of PSE-NHEKs in cultures of primary keratinocytes from twelve out of twelve different donors. A similar emergence from the senescence plateau has been observed with human mammary epithelial cells [28].

Each NHDF-conditioned medium (-CM) used in this study was obtained from 300,000 young or senescent NHDFs cultured in 6 ml FGM for 3 days, and was filtrated through a 0.45-µm-pore-size filter unit (Millipore). Henceforth, YF-CM refers to medium conditioned by young NHDFs and SF-CM to medium conditioned by senesent NHDFs.

Treatment of NHEK cultures with NHDF-CM was carried out by replacing 10% of the regular KGM with NHDF-CM. Such a treatment was applied throughout the culture and the NHDF-CM-containing keratinocytes medium renewed every 2 days.

The PSNE frequency was estimated as follows. Senescent NHEKs obtained under different culture conditions were plated at low density (10,000 cells per 100-mm dish). After 1 week, microscopy analysis revealed PSNE under all conditions tested, as the cell density remained low in the dish. Each dish was carefully scanned at least twice under the microscope, and for each dish the total number of PSE-NHEK clones, the number showing epithelioid morphology, and the number showing fibroblastoid morphology were recorded. The frequency of PSE-NHEK emergence was calculated as the ratio of the number of recorded clones to the number of initially seeded senescent cells.

The PAR-1 agonist peptide SFLLRN (RP19979) was from Genescript.

### Western Blotting

Western-blot analyses were performed on NHDF-conditioned media and on NHDF and NHEK lysates. CM was concentrated by ultrafiltration with a cut-off of 3 kDa (Amicon®, Millipore) and directly diluted in Laemmli buffer. The cells were lysed on ice in Laemmli loading buffer. Cell extracts and concentrated CM were boiled for 5 min. Gel loading was adjusted according to the number of cells lysed or used for CM preparation. Equal protein loading was checked *a posteriori* on the basis of the GAPDH or actin level determined for each condition. Proteins were resolved by SDS-PAGE and transferred onto nitrocellulose membranes (Hybond-C Extra, Amersham). The primary antibodies used were: rabbit anti-human E-cadherin (Santa Cruz), mouse anti-human vimentin (Santa Cruz), mouse anti-human TWIST-1 (AbCam), mouse anti-human PAR-1 (ATAP-2, Santa Cruz), mouse anti-human MMP-1 (Calbiochem), mouse anti-human MMP-2, mouse anti-human c-Met (Invitrogen, clone 3D4), mouse anti-human TGF-β1 (R&D System), rabbit polyclonal anti-human HGF-α (Santa Cruz, sc-7949)**,** mouse anti-human actin, and mouse anti-human GAPDH (Santa Cruz). Secondary antibodies were peroxidase-conjugated anti-mouse IgG and anti-rabbit IgG (Jackson ImmunoResearch Laboratories). Peroxidase activity was detected by enhanced chemiluminescence (Amersham Biosciences). For quantitative analysis of the results, the films were scanned (LAS 3000, Fujifilm) and the density of each band was estimated with the MultiGauge® software (Fujifilm). The density of each band was divided by the density of the GAPDH or actin band, and the obtained value was normalized with respect to that obtained under control conditions.

### Migration Assays

Assays were performed either with young NHEKs or PSE-NHEKs. Cells were starved in basal keratinocyte medium (KBM) for 16 h and then seeded in KBM onto Transwell® cell culture inserts with 8-µm pore size (Falcon) at a density of 30,000 cells per well (24-well format). Different attractant media were placed in the lower chambers: 100% KBM (control) or 90% KBM-10% FGM, 90% KBM-10% YF-CM, 90% KBM-10% SF-CM, or 90% KBM-10%, CM harvested from NHDFs treated with 12.5 µM GM6001 (Chemicon) or transfected with siRNA (see the specific paragraph for the protocol of siRNA transfection). Alternatively, activated recombinant MMP-1 or MMP-2 (R&D System), dissolved at 50 µM in FGM, or recombinant HGF-SF or recombinant TGF-β1 (Peprotech), dissolved at 10 ng/ml in KBM, was used as attractant. As recombinant MMP-1 and MMP-2 are provided as inactive pro-forms, they were activated prior to use in migration assays by incubation for 2 h at 37°C with 1 mM 4-aminophenylmercuric acetate (APMA, Sigma) in assay buffer (50 mM Tris pH 7.4, 10 mM CaCl_2_, 150 mM NaCl), as indicated by the supplier. When appropriate, NHEKs were pretreated for 15 min at 37°C with ATAP-2 PAR-1-blocking antibody (10 µg/ml, Santa Cruz Biotechnology), WEDE-15 PAR-1-blocking antibodies (20 µg/ml, Beckman Coulter), or both before seeding onto Transwell inserts. Alternatively, they were transfected with a control or PAR-1-specific siRNA two days before starvation in KBM (see below for the siRNA transfection protocol). Migration was allowed to proceed for 30 h at 37°C. Cells that had not migrated were then removed by scraping from the top face of each insert, while cells having migrated to the lower face were fixed in methanol and stained with Hoechst 33258 (40 ng/ml). Ten images of each filter were randomly captured with an Axioplan2 (Zeiss, Germany) microscope and an Axiocam HRc camera (Zeiss). Cells present on each image were counted with the Colony1.1® software.

### Reverse-transcription Quantitative Real-time PCR (RT-qPCR)

RNAs were isolated with the Nucleospin® kit (Macherey-Nagel). Reverse transcription was carried out for 1 h at 55°C with 1 µg total RNA, oligo dT primers, dNTPs and Superscript II reverse transcriptase (Invitrogen; 200 units). Primers for PCR (see Table S1) were designed with the NCBI-Primer-BLAST software (http://blast.ncbi.nlm.nih.gov/). The PCR protocol was as recommended for the Mx3005P Real-time PCR System® (Stratagene). Accumulation of PCR products was measured by SYBR green® fluorescence (SYBR Green® master mix; Applied Biosystems). Raw data analysis was performed with the MxPro® software (Agilent). For each sample, Ct(gene) - Ct(gapdh) was calculated, and this value was used to calculate the ratio of test gene mRNA to gapdh internal control mRNA.

### ELISA

Conditioned culture medium from 500,000 young or senescent NHDFs was harvested after 48 h of culture, filtered through a 0.45-µm-pore-size filter unit (Millipore), and concentrated by ultrafiltration with a cut-off of 3 kDa (Amicon®, Millipore). Quantification of HGF/SF was performed with the Quantikine® ELISA human HGF immunoassay (R&D Systems) according to the manufacturer’s instructions.

### In-gel Zymography Assays

Latent and active forms of MMP-1 and -2 secreted into the culture medium were assayed on the basis of their ability to digest gelatin, as described previously [29]. Briefly, proteins of fibroblast-conditioned media were resolved by SDS-PAGE under non-reducing conditions in 10% polyacrylamide gels containing 0.1% gelatin. Following electrophoresis, the gels were treated with zinc- and calcium-containing buffer to allow enzyme refolding. Gelatin digestion was then allowed to proceed for 24 h and the gels were finally stained with R-250 Coomassie blue. It should be noted that under these conditions of enzyme refolding and digestion of denatured gelatin (instead of native collagen), both the active and latent proteinase isoforms show activity. MMPs are identified on the basis of their respective molecular weights.

### Zymography Assays on Tissue Sections

Paraffin-embedded sections of skin biopsies from healthy young (26- to 38-year-old) and aged (60- to 89-year-old) human donors were dewaxed and rehydrated. Detection of gelatinolytic activity in the tissue sections was adapted from [30]. Briefly, DQ™ Gelatin from pig skin (1 mg/ml) (Molecular Probes), a fluorescein-conjugated gelatin in which fluorescence is quenched, was diluted (1/50) in reaction buffer (50 mM Tris-HCl pH 7,6, 150 mM NaCl, 5 mM CaCl_2_) and poured onto the surface of the tissue sections, covered with Parafilm® to avoid evaporation, and incubated in the dark for 4 h at 37°C in a humidified chamber. After removal of the Parafilm®, the sections were rinsed with Milli-Q water, fixed in 4% PFA for 10 min, and the nuclei were stained with Hoechst 33258 (Sigma-Aldrich) at 1 µg/ml for 5 min. Sections were finally mounted in Glycergel® mounting medium (Dako). The presence of active MMPs was revealed by the release of dequenched fluorescent peptides from the lower layer of gelatin.

### MMP-1, MMP-2, and PAR-1 Knockdown by RNA Interference

For each knockdown (of MMP-1 or MMP-2 in senescent NHDFs or of PAR-1 in PSE-NHEKs), a 20-nM pool of 4 targeting siRNAs was used (respectively: on-target plus SMARTpool® L-005951-00-0005, L-005959-00-0005, or L-005094-00-0005, Dharmacon). A non-targeting siRNA pool (siGENOME RISC-Free Control siRNA, Dharmacon) was used as control. siRNA transfections were performed with Lipofectamine™ RNAiMAX transfection reagent (Invitrogen) in opti-DMEM (Gibco). After 6 h of incubation at 37°C, the transfection medium was replaced with fresh culture medium. Conditioned media from the cultures of transfected cells were collected 72 h later and the cells lysed for protein or RNA extraction.

### Immunofluorescence Detection of PAR-1

NHEKs were seeded onto coverglasses and fixed with 2% paraformaldehyde in PBS. Antigen retrieval was carried out for 30 sec in citrate buffer (10 mM, pH 6) in a microwave oven set at 80 Watts. Nonspecific binding was blocked by incubation in PBS+2% BSA. The coverglasses were then incubated with mouse anti-human PAR-1 primary antibody (ATAP-2) (Santa Cruz Biotechnology). As a control, buffer was used instead of primary antibody. Rhodamine-Red-conjugated anti-mouse IgG (Jackson ImmunoResearch Laboratories) was used as secondary antibody. Nuclei were stained for 5 min with Hoechst 33258 (Sigma-Aldrich) at 1 µg/ml and the coverglasses mounted in Glycergel® mounting medium (Dako). Optical sectioning images were taken with an Axioplan2 (Zeiss, Germany) microscope equipped with an Apotome device and either a Plan Neofluar (5x, NA: 0.16), an Apochromat (20x, NA:0.8), or a Plan Neofluar (40x, NA: 1.3) objective. Images (12 bits, 1388/1040 pixels, no binning) were recorded with an Axiocam HRc camera (Zeiss) (3200°K) and AxioVision® Software was used for microscope image analysis (Zeiss). Images to be combined in a panel shared the same initial properties and gamma, and were exported as 8-bit TIFF files. They were subjected to the same software brightness and contrast adjustments in PowerPoint®.

### Immunohistochemical Detection of PAR-1 in Tissue Sections

Deparaffinized, rehydrated sections of skin biopsies from healthy young (26- to 38-year-old) or aged (60- to 89-year-old) human donors, obtained from the Bonn University (Germany) tumor library, were treated with 1% H_2_O_2_ in PBS to block endogenous peroxidases. Nonspecific binding was prevented by incubation in PBS+5% BSA and 10% rabbit non-immune serum. Endogenous avidin and biotin were inhibited with the specific Avidin/Biotin Blocking Kit of Vector Labs. Primary antibodies (monoclonal mouse anti-PAR-1, Santa Cruz Biotechnology, 1/100) and secondary antibodies (biotinylated rabbit anti-mouse, Dako, 1/100) were diluted, respectively, in PBS+5% BSA+10% rabbit non-immune serum or PBS+2% BSA. An isotype-specific control immunoglobulin (Santa Cruz Biotechnology) was used on an adjacent section. Detection was done with streptavidin-peroxidase (Jackson Immunoresearch) and the DAB Peroxidase Substrate Kit (Vector Labs). Slices were counterstained with Gill’s hematoxylin in tap water and mounted in Dako Glycergel®.

## Results

### Soluble Factors Secreted by Senescent NHDFs Promote PSNE and EMT of PSE-NHEKs

To test whether factors secreted by senescent NHDFs might promote PSNE of NHEKs, we compared the growth and evolution of NHEKs cultured in either regular medium (KGM) or 90% KGM-10% YF-CM, SF-CM, or fresh FGM. In each case the NHDFs used to prepare the CM and the CM-treated NHEKs were autologous, and the CM was included throughout NHEK culture. The proportion of CM in the culture medium was only 10% in volume, so as to limit the final serum concentration to 0.2%, a level low enough to avoid NHEK differentiation. As previously described [26], NHEKs cultured in KGM entered senescence after 10–15 PDs, remained at the plateau for a few days, and then a fraction of senescent NHEKs underwent a peculiar budding mitosis mechanism generating small, transformed PSE-NHEKs, which pursued clonal growth. Supplementation with FGM, YF-CM, or SF-CM strongly promoted PSE-NHEK growth (Figure 1A). Since this effect was identically induced by all FGM-containing media, it was attributed to serum. We also evaluated the effects of CM on the PSNE frequency. For this, senescent NHEKs obtained in the different culture media were plated at low density and monitored for PSNE, which occurred after a week in all culture media. PSE-NHEK clones were counted under careful microscopic observation. Cultures in FGM- or YF-CM-supplemented KGM showed only a slight, non-significant increase in PSNE frequency as compared to KGM alone, whereas the SF-CM-supplemented culture showed a 2-fold increase (Figure 1B and Figure S2A). Remarkably, while PSE-NHEKs clones formed under control conditions grew in islets and displayed an epithelioid morphology, almost all PSE-NHEK clones produced in the presence of SF-CM consisted of scattered cells with fibroblastoid morphology (as depicted at different magnifications in Figure 1B). In cultures containing FGM or YF-CM, both epithelioid and fibroblastoid PSE-NHEK clones were present (Figure 1B and Figure S2A). Because of these differences, the PSE-NHEKs obtained in FGM-, YF-CM- and SF-CM-supplemented medium are henceforth called PSE-NHEK/FGM, PSE-NHEK/YF-CM, and PSE-NHEK/SF-CM cells, respectively.

The observed shift from an epithelioid to a fibroblastoid morphology in the presence of SF-CM suggests that PSE-NHEK/SF-CM cells have undergone EMT. To test this interpretation, we first looked for a change in EMT marker levels. Western-blot analysis indicated that PSE-NHEK/SF-CM cells have a dramatically reduced level of the epithelial marker E-cadherin and an increased level of the fibroblast marker vimentin. TWIST-1, a transcription factor involved in EMT induction, was also present at a higher level in PSE-NHEK/SF-CM cells than in PSE-NHEK/FGM or PSE-NHEK/YF-CM cells (Figure 1C and Figure S2C for quantitative data). Although CM was supplied throughout kerotinocyte culture, the E-cadherin and vimentin levels were affected only in PSE-NHEKs and not in young NHEKs (Figures S2B and S2C). TWIST-1 was not detected in young NHEKs (data not shown).

Acquisition of migratory ability being a hallmark of EMT [31], we used Transwell® assays to compare the migratory abilities of PSE-NHEKs obtained in the different CM-containing culture media. We also compared the attractant power of the different culture media when placed in the lower chambers of the Transwells. Migration of control PSE-NHEK/KGM cells was very modest, whatever the attractant. In contrast, PSE-NHEK/FGM, PSE-NHEK/YF-CM, and PSE-NHEK/SF-CM cells showed significant migration upon stimulation. PSE-NHEK/SF-CM cells migrated more than the others (Figure 1D), confirming that these cells had undergone EMT. All three PSE-NHEK types responded more strongly to SF-CM than to YF-CM or FGM (Figure 1D and Figure S2D for statistical analyses), indicating that SF-CM contains soluble molecules having very strong pro-migratory effects. Very interestingly, PSE-NHEK/SF-CM cells responded more strongly to SF-CM than did PSE-NHEK/YF-CM cells, whereas both cell types responded equally to YF-CM and FGM (Figure 1D and Figure S2D). This suggests that long-term exposure to SF-CM upregulates in NHEKs the synthesis and/or activation of receptors, thus sensitizing them to pro-migratory factors specifically secreted by senescent fibroblasts.

### Neither HGF/SF nor TGFβ-1 is Responsible for SF-CM-induced Migration of PSE-NHEKs

Our next objective was to identify ligand-receptor signaling axes that might contribute to mediating the pro-migratory effects of SF-CM on PSE-NHEK/SF-CM cells. We first used RT-qPCR to screen for transcripts encoding proteins previously described as components of the senescent secretome [10], [32] or known to participate in EMT [33]. We thus confirmed upregulated levels of several cytokine (MCP-1, IL-1, IL6, GRO-1) and growth-factor transcripts (TGF-β1, HGF/SF, VEGF and AREG) in senescent NHDFs *versus* young ones (Figure S3A and B).

Amongst the factors showing enhanced transcript levels in senescent NHDFs, we first focused on HGF/SF because of its cell scattering function [34] liable to contribute to the scattered phenotype of PSE-NHEK/SF-CM clones and to favor cell migration. We first made sure that HGF/SF is oversecreted by senescent NHDFs, by performing western-blot and ELISA analyses of the culture media (Figure S4A and B). We then examined in Transwell® assays whether recombinant HGF/SF might stimulate migration of PSE-NHEKs. HGF/SF proved able to stimulate slight migration of both young NHEKS (control) and PSE-NHEKs (Figure S4C) However, HGF/SF was unable to reproduce the strong attractant effect of SF-CM on PSE-NHEK/SF-CM cells (Figure S4D), indicating that PSE-NHEK/SF-CM cells are not sensitive to HGF/SF. In support of this result, PSE-NHEKs showed a reduced to undetectable level of c-met transcripts (c-Metis the receptor of HGF/SF) and complete loss of C-Met protein when cultured in the presence of any CM (Figure S4E and F). Taken together, these results rule out a direct involvement of HGF/SF-c-Met signaling in SF-CM-induced migration of PSE-NHEK/SF-CM cells.

We then evaluated the role of TGF-β1, a central inducer of EMT [35] associated with early cancer dissemination [36]. We checked by western blotting that the active form of TGF-β1 is oversynthesized in senescent NHDFs as compared to young ones (Figure S4H). We then examined whether recombinant TGF-β1 might trigger migration of PSE-NHEK/SF-CM cells. In Transwell assays, TGF-β1 used as attractant proved unable to stimulate PSE-NHEK/SF-CM migration (Figure S4D). Furthermore, the level of TGF-βRII receptor transcripts showed no clear variation according to the growth stage or culture conditions (Figure S4G). It thus appears that the TGF-β1-TGFβRII axis is not directly involved in the response of PSE-NHEK/SF-CM cells to the promigratory action of SF-CM.

### MMP-1 and -2 Secreted by Senescent Skin Fibroblasts Induce Migration of PSE-NHEKs

Beside cytokines and growth factors, MMPs have been reported as major components of the secretomes of senescent prostate, breast, and skin fibroblasts [10], [37], [38]. Their proteinolytic action on extracellular matrix components makes them major actors of cancer cell invasion and metastasis. However, MMPs have also cell surface targets that can mediate EMT [39], [40].These facts led us to examine whether MMPs might drive the SF-CM-induced migration of PSE-NHEKs.

**Figure 2:**
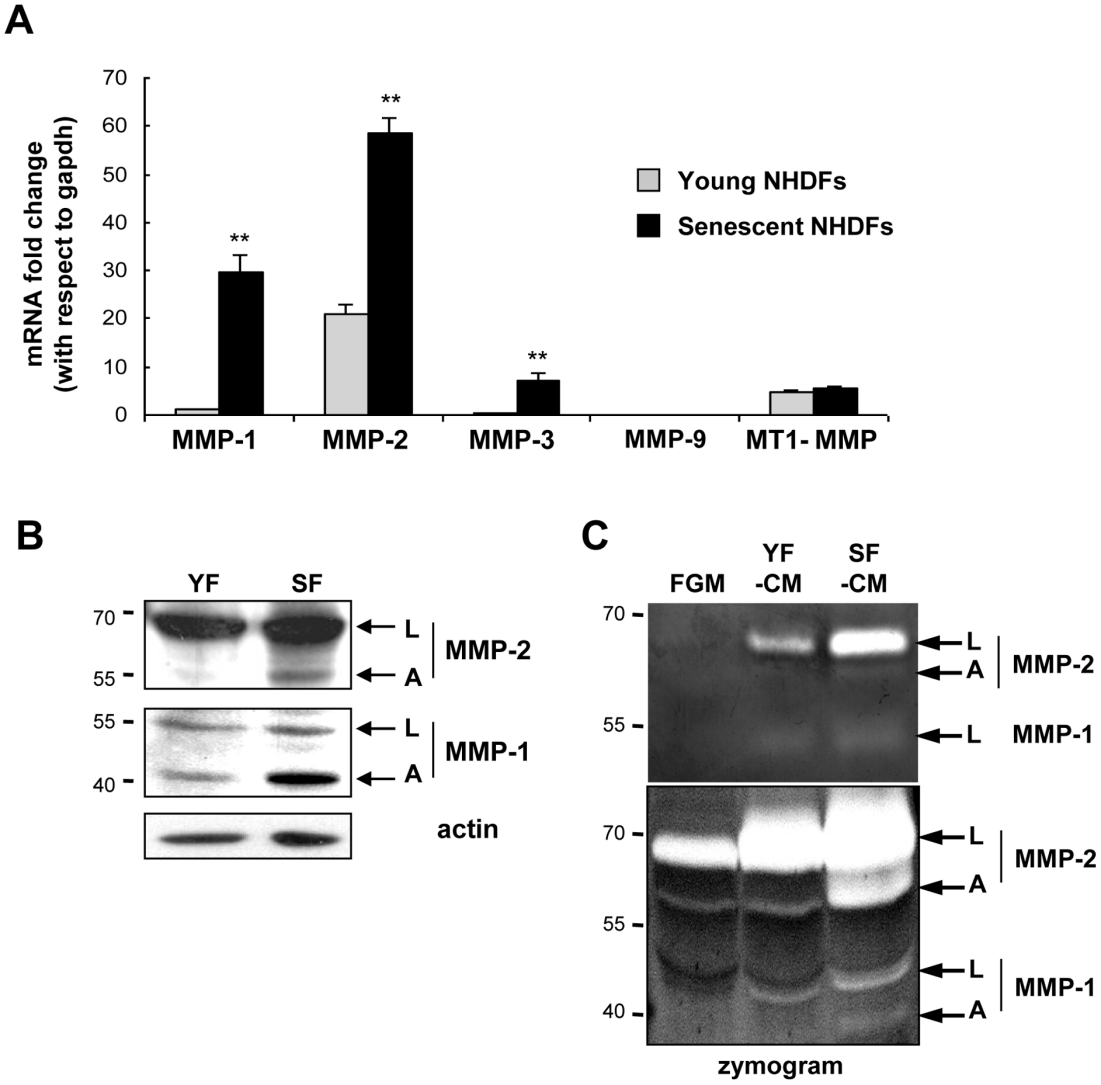
MMP- 1 and -2 are oversynthesized, oversecreted, and overactivated by senescent dermal fibroblasts. **A.** RT-qPCR analysis of MMP-1, MMP-2, MMP-3, MMP-9, and MT1-MMP transcripts in young and senescent NHDFs. MMP-9 transcripts were undetectable. Results are means of quadruplicates ± SD. Data are representative of 5 independent experiments performed with NHDFs from donors 2F1966 and 1619 (**: p<0.01). **B.** Western-blot analysis of MMP-1 and MMP-2 in lysates of young (YF) and senescent (SF) NHDFs. L and A indicate, respectively, the latent and active forms of the MMPs. Actin was used as as loading control. Quantitative analysis is provided in Figure S5A. Data are representative of two independent experiments with NHDFs from donor 2F1966 and were similar to results obtained with cells from other donors. **C.** Analysis by in-gel zymography of the proteinolytic activity of MMP-1 and MMP-2 secreted by young and senescent NHDFs and present in the conditioned media (respectively YF-CM and SF-CM). FGM was used as a control for basal MMP activities present in serum. The analysis was done by SDS-PAGE with a gel containing 0.1% gelatin. Either 5 µl (upper part) or 20 µl conditioned medium was loaded. White bands denote the presence of proteins with gelatinolytic activity. L and A indicate, respectively, the latent and active forms of the MMPs. Data are representative of 6 independent experiments performed with cells from donors 2F1966 and 1619.

We first used RT-qPCR to compare levels of MMP transcripts in senescent *versus* young NHDFs. Levels of both MMP-1 and MMP-2 transcripts appeared strongly upregulated in senescent NHDFs. MMP-3 transcripts were also upregulated at senescence, although to a lesser extent than MMP-1 and MMP-2 transcripts. MMP-9 transcripts were undetectable in both young and senescent NHDFs. The level of MT1-MMP transcripts was very low and did not increase at senescence (Figure 2A). On the basis of these results we focused on MMP-1 and -2 protein levels. Senescent NHDFs showed higher levels than young NHDFs of both the latent and active (cleaved) forms of MMP-1 and -2 (Figure 2B and Figure S5A and B). By in-gel zymography, activated MMP-1 and -2 were detected in SF-CM but not in YF-CM or FGM, and higher levels of inactive forms were also found in SF-CM (Figure 2C). In addition, a dramatically lower level of TIMP-1 (Tissue Inhibitor of Metalloproteinase 1) was found in SF-CM than in YF-CM (Figure S5C and D).

**Figure 3:**
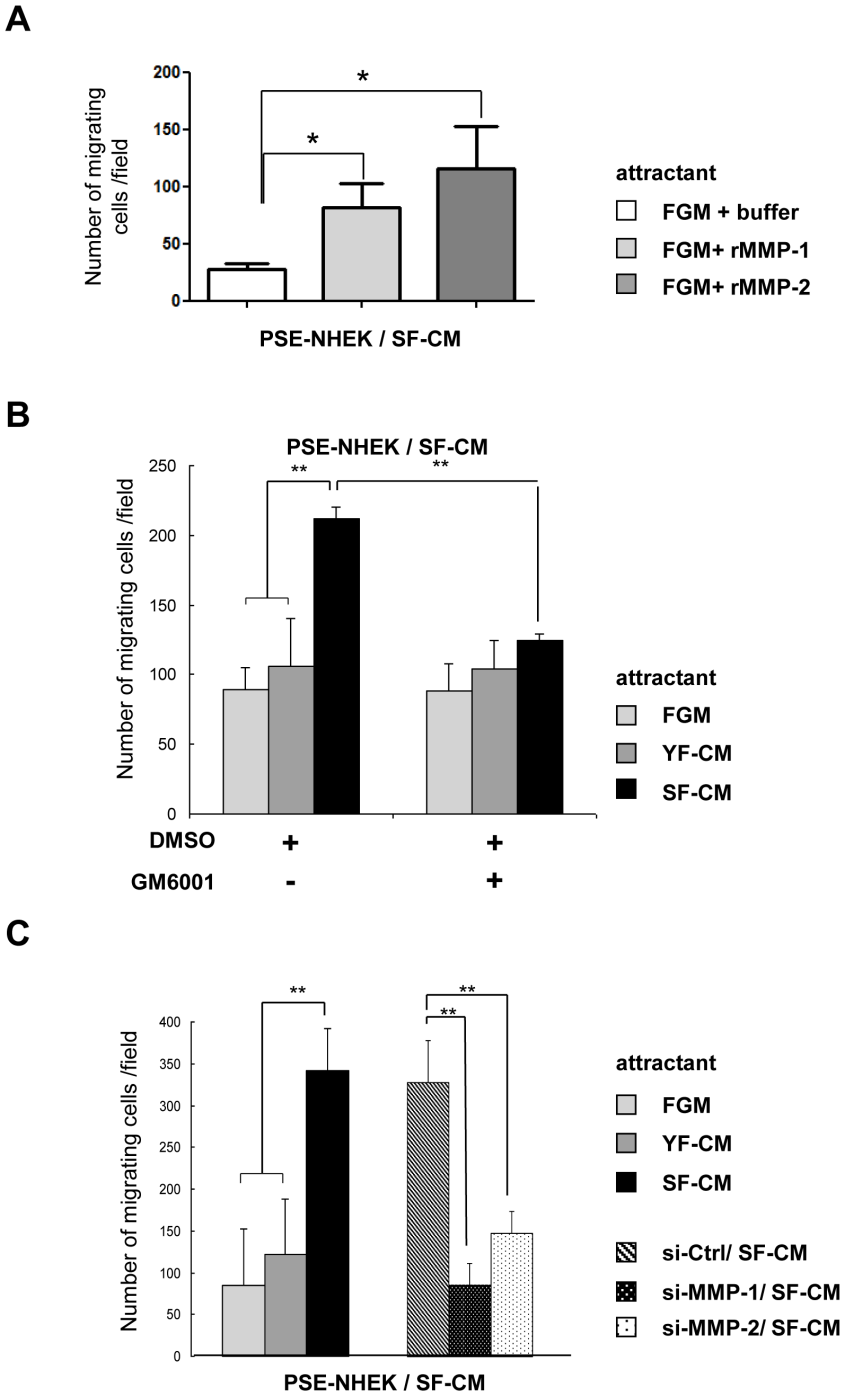
MMP-1 and -2 in senescent-dermal-fibroblast-conditioned medium stimulate PSE-NHEK migration. Migration assays in Transwell® chambers. PSE-NHEK/SF-CM cells (donor 2F1958) were starved in fresh KBM medium and seeded in KBM onto the tops of the inserts, and various attractant media were put in the lower chamber. **A.** Recombinant activated MMP-1 or MMP-2 was used at 50 µM in FGM as attractant medium. **B.** The MMP inhibitor GM6001 (12.5 µM) or DMSO was added to FGM, YF-CM, or SF-CM that was subsequently used as attractant medium. **C.** Senescent fibroblasts were transfected with non-targeting control siRNAs or with MMP-1 or MMP-2 siRNAs. After 72 h, the conditioned media were collected and used as attractant media. In each experiment, cells having migrated were counted in ten random fields per well, with condition in triplicate. Results are given as means ±SD (*: p<0.05; (**: p<0.01). Data are representative of two (in A) or three (in B and C) independent experiments.

We then tested whether PSE-NHEK/SF-CM migration might be stimulated by active recombinant MMP-1 or MMP-2. Transwell® assays revealed that both metalloproteinases can promote a strong migratory phenotype in this cell population (Figure 3A). To formally establish that MMPs contribute to the pro-migratory action of SF-CM on PSE-NHEK/SF-CM cells, we used GM6001, a broad-spectrum inhibitor of MMP proteinolytic activity. First we tested the efficacy of GM001 against MMP-1 and -2 in zymography assays (Figure S6A). Then the inhibitor was added to FGM, YF-CM, and SF-CM prior to their use as attractants in Transwell® assays. GM6001 treatment was found to decrease the pro-migratory effect of SF-CM to the level observed with FGM or YF-CM (Figure 3B). To confirm this result and to compare the contributions of MMP-1 and -2 to the pro-migratory action of SF-CM, we specifically knocked down MMP-1 or MMP-2 expression with siRNAs in senescent NHDFs, harvested the corresponding conditioned media, and evaluated the ability of each conditioned medium to attract PSE-NHEK/SF-CM cells. The efficacy of the siRNAs was checked by in-gel zymography (Figure S6B). Like GM6001 treatment, specific knockdown of either MMP-1 or MMP-2 in NHDFs abolished the pro-migratory effect of the corresponding SF-CM. Both proteinases thus seem to contribute to the attractant effect (Figure 3C). We conclude that active MMP-1 and MMP-2 oversecreted and overactivated by senescent fibroblasts are sufficient to induce PSE-NHEK migration, and that proteinolytic activity is required for this process.

### PAR-1 is Specifically Expressed by PSE-NHEKs and Mediates Stimulation of PSE-NHEK Migration by MMP-1 and MMP-2

We then investigated which receptor might mediate the pro-migratory effects of MMP-1 and/or MMP-2. A bibliographical survey highlighted PAR-1 (Protease-Activated Receptor-1), a G-protein-coupled receptor whose activation results from the irreversible proteinolytic cleavage of its amino-terminal exodomain, exposing a tethered ligand [41]. This proteinolytic activation can be achieved by several proteases, including MMP-1, thrombin, coagulation factor Xa, granzyme A, and activated protein C [42]. MMP-1 has been shown behave like thrombin, enhancing the migration of highly invasive breast cancer cells through proteinolytic activation of PAR-1 [43]. Moreover, PAR-1 activation appears associated with keratinocyte migration [44], [45].

**Figure 4:**
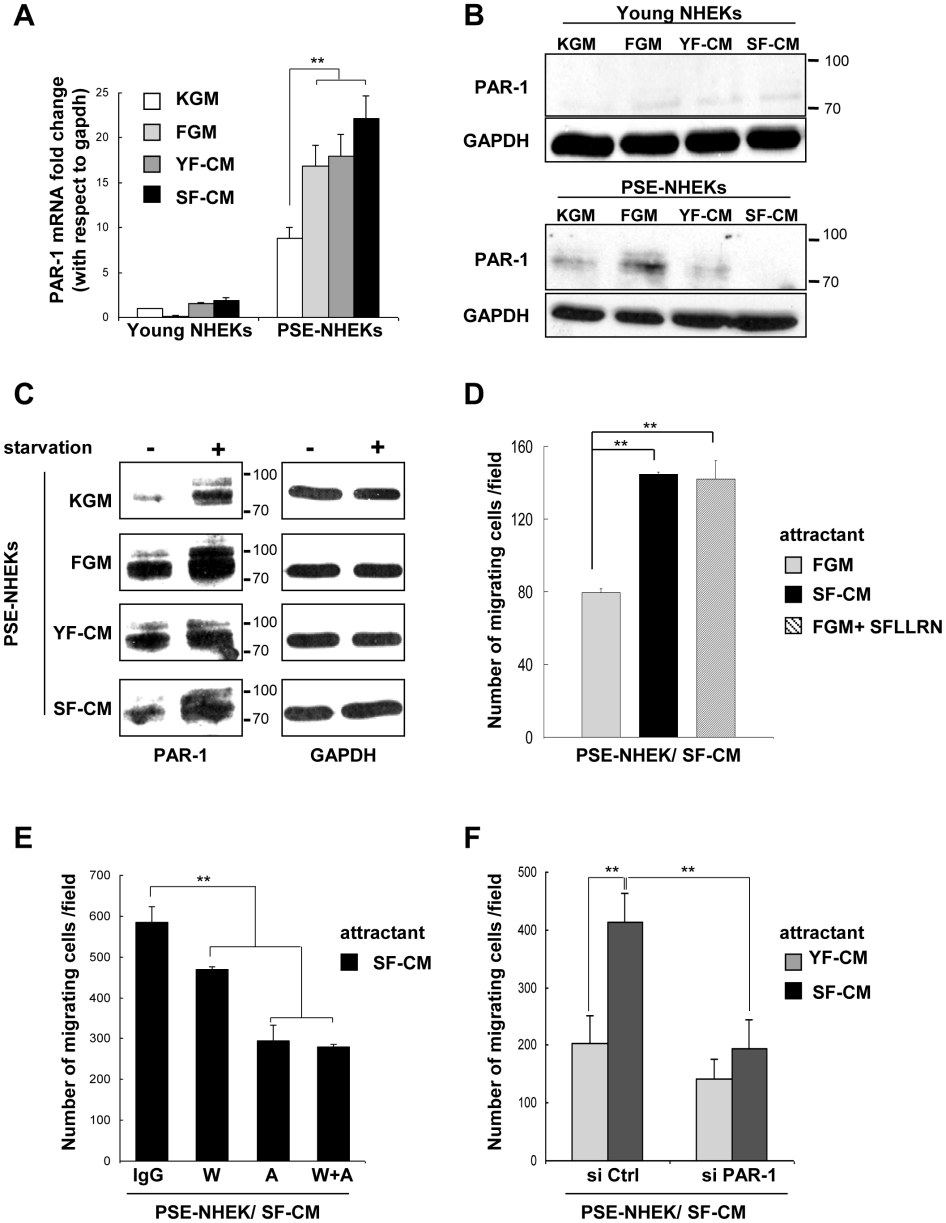
The PAR-1 receptor is oversynthesized in PSE-NHEK/SF-CM cells and mediates stimulation of migration by SF-CM. **A.** RT-qPCR analysis of PAR-1 transcripts in young- and PSE-NHEK (donor 2F1958) cultured under the different conditions described in figure 1A (KGM, FGM, YF-CM, or SF-CM). Results are means of triplicates ±SD (**: p<0.01). Data are representative of 3 independent experiments. **B.** In parallel, PAR-1 protein (80 kDa) was analyzed by western blotting in the various young NHEKs and PSE-NHEKs. GAPDH was used as loading control and quantitative analysis of PAR-1 levels is provided in Figure S7A. **C.** Western-blot analyses of PAR-1 protein in the different PSE-NHEKs starved or not in fresh KBM. Data are representative of two independent experiments. GAPDH was used as loading control and quantitative analysis is shown in Figure S7B. **D**. Migration assays in Transwell® plates. PSE-NHEK/SF-CM cells (donor 2F1958) were starved in fresh KBM (i. e. non-supplemented basal keratinocyte culture medium) for 16 h, trypsinized, and seeded onto Transwell® inserts. FGM, SF-CM, or FGM supplemented with the SFLLRN PAR-1 agonist peptide (100 µM) were placed in the lower chamber as attractant and cells were allowed to migrate for 30 h. **E.** Migration assays in Transwell® plates. PSE-NHEK/SF-CM cells (donor 2F1958) were starved in fresh KBM, trypsinized, treated with WEDE-15 PAR-1-blocking antibody (W; 20 µg/ml), ATAP-2 PAR-1-blocking antibody (A; 10 µg/ml), both blocking antibodies combined (W+A), or control IgG, seeded in KBM, and allowed to migrate for 30 h. SF-CM was used as attractant. **F**. Migration assays in Transwell® plates. PSE-NHEK/SF-CM cells (donor 2F1958) were transfected with a pool of 4 non-targeting control siRNAs (si Ctrl) or a pool of PAR-1 siRNAs (si PAR-1). After 72 h, the cells were starved in KBM for 16 h, trypsinized, seeded in KBM, and allowed to migrate for 30 h. YF-CM or SF-CM were used as attractants. The experiment was also done in parallel with PSE-NHEK/FGM and PSE-NHEK/YF-CM. The results are given in Figure S6D. **D–F**. Cells having migrated were counted in ten random fields per well, and each condition was tested in triplicate. Results are given as means ± SD (**: p<0.01). Similar results were obtained in two (for E) or three (for F) independent experiments.

We first examined whether PAR-1 is upregulated in PSE-NHEK/SF-CM cells. RT-qPCR revealed that PAR-1 transcripts are strongly increased in control PSE-NHEKs as compared to young NHEKs, and more strongly (albeit similarly) increased in PSE-NHEK/FGM, PSE-NHEK/YF-CM, and particularly in PSE-NHEK/SF-CM cells (Figure 4A). Western-blot analysis of PAR-1 (80 kDa) confirmed an increased PAR-1 level in control PSE-NHEKs as compared to young NHEKs, but surprisingly, PSE-NHEK/SF-CM cells consistently showed a lower level of PAR-1 protein than PSE-NHEK/FGM or PSE-NHEK/YF-CM cells (Figure 4B and Figure S7A). It is established that, once activated by proteinolytic cleavage, PAR-1 receptors are rapidly internalized and targeted to lysosomes for degradation [46]. Hence, we speculated that under the continuous influence of SF-CM, the cell surface pool of PAR-1 receptors might be continuously activated and degraded. To test this hypothesis, the different PSE-NHEK populations were starved in fresh non-supplemented KBM. This restored a pool of full-length PAR-1 in each PSE-NHEK population (Figure 4C and Figure S7B), and the levels of these pools correlated well with transcript levels (Figure 4A). PAR-1 thus emerged as a candidate mediator of the migratory effects of SF-CM.

To further evaluate the involvement of PAR-1 in mediating the pro-migratory effect of SF-CM, PSE-NHEK/SF-CM migration was tested in Transwell® assays where SFLLRN, the minimal tethered-ligand-mimicking PAR-1 agonist peptide [46], [47], was used as attractant. The PAR-1 agonist completely mimicked the pro-migratory effect of SF-CM (Figure 4D), showing that PAR-1 activation is sufficient to induce migration of PSE-NHEK/SF-CM cells. Conversely, blocking of PAR-1 activation with blocking antibodies (ATAP-2 or WEDE-15) strongly reduced the migratory response of PSE-NHEK/SF-CM cells to SF-CM (Figure 4E). Similarly, specific PAR-1 knockdown in PSE-NHEK/SF-CM cells by siRNAs (see Figure S6C showing siRNA efficacy) completely abolished the pro-migratory effect of SF-CM (Figure 4F and Figure S6D), demonstrating the involvement of PAR-1 in mediating the pro-migratory effect of SF-CM.

**Figure 5:**
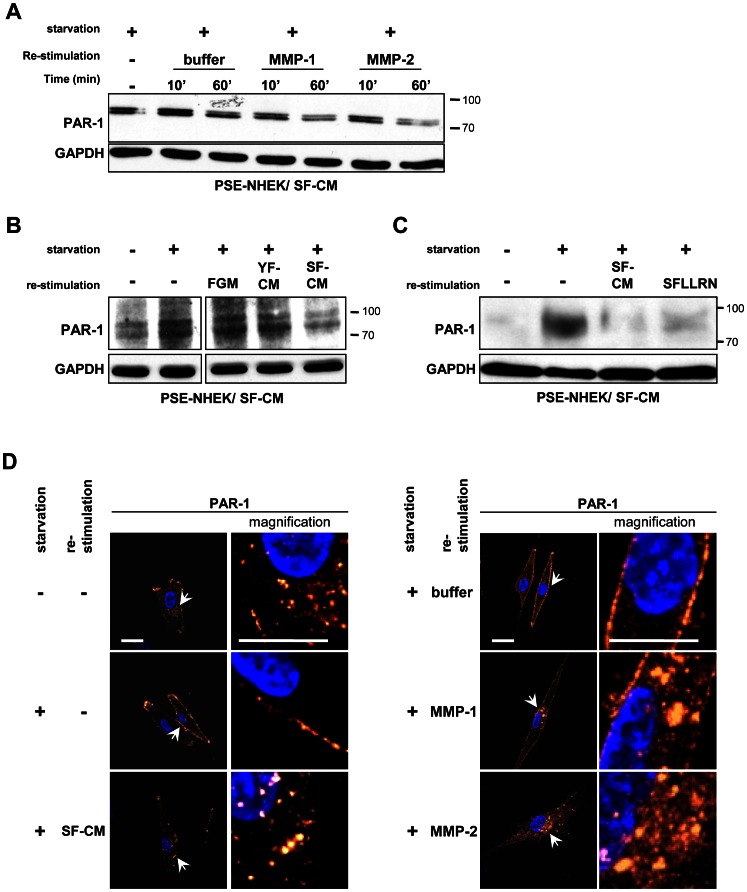
PAR-1 is activated by both SF-CM and recombinant MMP-1 and -2, internalized, and degraded in lysosomes. PSE-NHEK/SF-CM cells (donor 2F1958) were starved in fresh KBM for 16 h to replenish the PAR-1 pool (non-activated and thus undegraded). They were then incubated with: **A**. activated recombinant MMP-1 or MMP-2 (50 µM) or control buffer for 10 min or 1 h; **B.** medium containing 10% fresh FGM, YF-CM, or SF-CM for 1 h; **C.** medium containing 10% SF-CM or the peptide agonist of the PAR-1 receptor (SFLLRN, 100 µM) for 1 h. The cells were then lysed for western-blot analysis of PAR-1. GAPDH was used as loading control. Each figure is representative of two independent experiments. A–C, Quantitative analyses are given in Figure S7C-E. D. PSE-NHEK/SF-CM cells were seeded onto coverglasses, starved in fresh KBM for 16 h, incubated for 1 h in 10% SF-CM, recombinant MMP-1 and MMP-2, or MMP control buffer, fixed, and processed for immunofluorescence detection of PAR-1. Each condition was tested in triplicate; representative images are shown. Arrows indicate the location of images taken at higher magnification. Scale bar  = 10 µm. Results are representative of two independent experiments.

To further test the above conclusion, we investigated whether PAR-1 expressed by NHEK/SF-CM cells can be activated by MMP-1, MMP-2, or SF-CM. For this, PSE-NHEK/SF-CM cells were starved so as to restore the pool of full-length non-activated PAR-1. The cells were then subjected for 10 or 60 min to active recombinant MMP-1 or MMP-2. Western blotting showed that treatment with either MMP for 60 minutes led to re-degradation of the receptor (Figure 5A and Figure S7C for quantitative data). Alternatively, starved PSE-NHEK/SF-CM cells were treated with SF-CM, YF-CM, or FGM for one hour. Incubation with SF-CM led to re-degradation of the receptor, whereas incubation with either YF-CM or FGM did not (Figure 5B and Figure S7D). To make sure that PAR-1 degradation was indicative of its activation, we compared the effect of SF-CM with that of the PAR-1 agonist peptide SFLLRN, known to induce PAR-1 activation, internalization by endocytosis, and final degradation inside lysosomes [46], [47]. Incubation of starved PSE-NHEKs with SFLLRN or SF-CM led to similar degradation of the receptor (Figure 5C and Figure S7E). The subcellular localization and trafficking of the receptor after stimulation was then analyzed by immunofluorescence. In non-starved, unstimulated PSE-NHEK/SF-CM cells, the receptor was found both at the cell surface and inside the cytoplasm. In starved, unstimulated cells, the receptor was restricted to the cell surface. In starved cells stimulated by MMP-1, MMP-2, or SF-CM, the receptor was detected inside cytosolic vesicles (Figure 5D). PAR-1 was also present mostly at the cell surface of starved PSE-NHEK/YF-CM cells, but their re-stimulation by YF-CM did not induce any significant change in PAR-1 localization (Figure S8). Treatment with bafilomycin A1, an inhibitor of the H+ ATPase and of degradative lysosomal activity [48], led to a greater accumulation of PAR-1 in cytosolic vesicles upon re-stimulation with SF-CM, confirming the lysosomal destination of activated PAR-1 (Figure S8). Altogether, these results demonstrate that SF-CM, MMP-1, and MMP-2 can engage the normal activation pathway of the PAR-1 receptor in PSE-NHEK/SF-CM cells.

### MMPs and PAR-1 are Present in Aged Skin and Skin Dysplasias, but not in Young, Healthy Skin Samples

**Figure 6:**
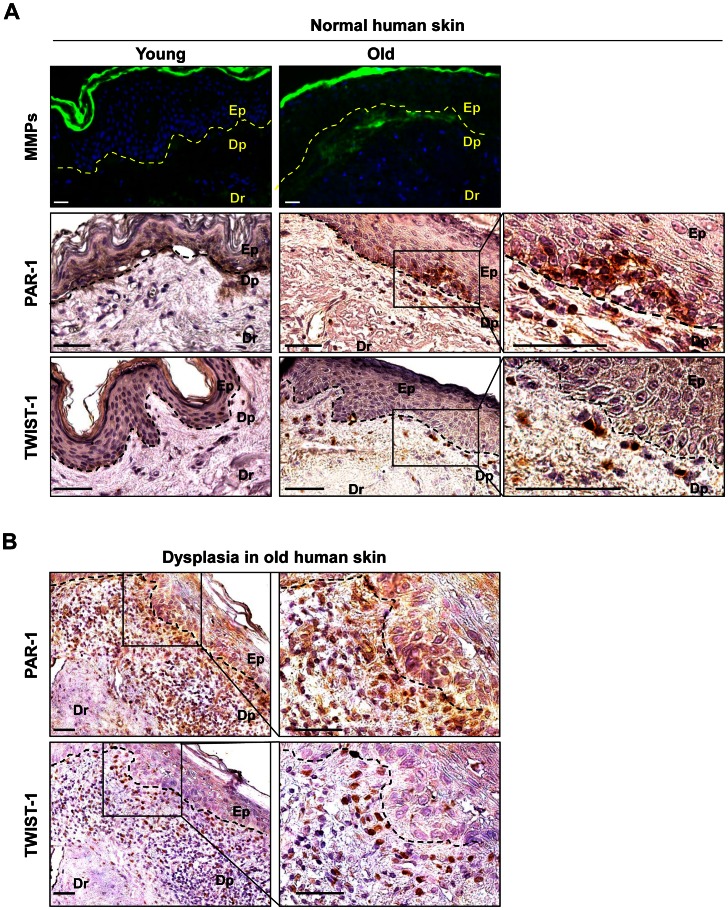
Detection of MMP gelatinolytic activity and of PAR-1 and TWIST-1 in human skin samples. **A.** Upper panel: Zymography assay on histological sections of skin from young (donor n°32645/09, 34 y.o.) or aged (donor n°28010/09, 89 y.o.) healthy human donors. MMP gelatinolytic activity was detected by fluorescence after cleavage of a quenched fluorescein-conjugated gelatin substrate. Ep: epidermis; Dp: papillary dermis; Dr: reticular dermis; (**-** **-** **-**) basal lamina. Bar = 25 µm. Specific fluorescence is detected mainly in the Dp. Similar results were obtained with material from other donors (three young and five aged) (see Table S2A and B). Middle and lower panels**:** Immunohistodetection of the PAR-1 and TWIST-1 proteins in healthy skin samples from a young donor (n°32645/09, 34 y.o.) and an aged donor (n°28010/09, 89 y.o.). Bar = 50 µm. Rectangles delimit images of higher magnification. PAR-1 is detected only in aged donors, in foci of disorganized basal keratinocytes, and in isolated cells in the underlying Dp. TWIST-1 is detected only in isolated cells in the Dp resembling those which are PAR-1-positive. See Table S2, A and B for results obtained with other donors. **B.** Immunohistodetection of the PAR-1 and TWIST-1 proteins in serial sections of skin samples from an aged donor with dysplasia (donor n°35772, 76 y.o.). Bar = 50 µm. The square delimits the image of higher magnification. PAR-1 and TWIST-1 are not detected in the hyperplastic epidermis itself, but in cells disseminated in the hypertrophic inflammatory and angiogenic Dp. Similar results were obtained with other donors and are summarized in Table S2C.

The above *in vitro* analyses show that PSE-NHEKs display numerous PAR-1 receptors, which are activated in the presence of MMPs secreted by senescent fibroblasts. This leads to greater migratory activity, particularly of PSE-NHEKs having arisen from long-term cultures with SF-CM. We next investigated the *in vivo* relevance of these results by comparing MMP and PAR-1 expression in healthy skin sections from aged *versus* young human donors and in sections of non-melanoma skin dysplasias (Figure 6). MMP activity was assayed in histological skin sections by fluorescence zymography. Gelatinolytic activity was higher in skin samples from aged donors than in samples from young ones. It was especially high in the papillary dermis, i. e. the part of the dermal tissue just below the basal lamina (Figure 6A), in the area closest to keratinocytes potentially sensitive to activating signals. These results confirm those of other investigators showing that aging of dermal fibroblasts is associated with increased MMP secretion, especially within the papillary dermis [38]. A weak signal was also recorded in the epidermis (Figure 6A), possibly reflecting either MMP secretion by senescent keratinocytes as described by others [49] or MMP diffusion from the dermis.

We then investigated PAR-1 expression in the epidermis by immunohistochemistry. No PAR-1 was detected in skin samples from young donors (five out of five donors) (Figure 6A and Table S2A for individual data). In aged donors, PAR-1 was occasionally detected in a subset of keratinocytes located in foci inside the basal layer of the epidermis (three out of seven donors) (Figure 6A and Table S2B). In these foci, the epithelial organization appeared disturbed, with loss of cell alignment and loosened intercellular cohesion. This observation suggests that a PSNE-like phenomenon might occur *in vivo* with aging, generating keratinocytes expressing PAR-1. Some PAR-1-positive cells were also observed inside the papillary dermis, just below the PAR-1-positive epidermal foci. Such PAR-1-positive cells might be PAR-1-positive keratinocytes having emigrated from the epidermal foci under the influence of increased MMP activity in the neighboring dermis. To test whether keratinocytes having undergone an EMT can migrate and enter the dermis, we performed TWIST-1 immunodetection. No TWIST-1 was detected in skin sections from young donors (five out of five donors) (Figure 6B and Table S2A). In aged donors, TWIST-1 was occasionally detected in cells scattered inside the papillary dermis (Figure 6A), suggesting that some keratinocytes could undergo an EMT during aging. Only four samples out of seven from aged donors displayed such TWIST-1-positive cells dispersed in the papillary dermis, but interestingly, all these samples also displayed dispersed PAR-1-positive cells in the same area of the papillary dermis (Table S2B).

If the dispersed cells overexpressing PAR-1in the papillary dermis resemble the PSE-NHEKs obtained *in vitro*, they should be transformed and able to proliferate, i. e. they might constitute the origin of dysplasia. We therefore investigated the expression of PAR-1 and TWIST-1 in sections of non-melanoma skin dysplasia. Numerous TWIST-1- and PAR-1-positive cells were detected in the dysplastic areas, in four out of four donors tested (Figure 6B and Table S2). Positive cells were generally not found in the dysplastic epidermis itself, but scattered through the underlying inflammatory and angiogenic papillary dermis (Figure 6B).

From these observations taken together, one can speculate that a PSNE-like phenomenon might occur *in vivo* with aging, generating transformed keratinocytes displaying a very early EMT.

## Discussion

It is well established that senescent fibroblasts can promote, both *in vitro* and *in vivo*, the malignant progression of already-transformed epithelial cells through secretion of inflammatory mediators [10], [11], [32], [50]. We show here that the secretome of senescent fibroblasts also has an impact on the first stages of carcinogenesis occurring by senescence evasion.

We have shown in a previous work that partially transformed, tumorigenic keratinocytes, here called PSE-NHEKs, systematically and spontaneously emerge from senescent cultures of primary human epidermal cells. This mechanism of senescence evasion is triggered by the mutagenic action of reactive oxygen species accumulating at senescence. PSE-NHEKs display transcriptomic and proteomic modifications indicative of an only partial transformed state, accompanied by a very slight EMT. Yet they can develop into many small, disseminated skin hyperplasias and carcinomas when xenografted into *nude* mice, indicating that they have actually undergone profound neoplastic changes [26]. In the present study, we establish that the generation of these cells and the modifications they undergo are influenced by the pro-tumorigenic switch of the aging microenvironment. We show that when exposed to a senescent fibroblast secretome, keratinocytes escape from senescence more frequently, and that the post-senescent keratinocytes display a stronger EMT, including loss of E-cadherin, upregulation of vimentin and TWIST-1, a scattered phenotype, and acquisition of a migratory capacity, these being very early events in carcinogenesis. In our previous study, we noticed that hyperplasias and carcinomas arising from xenografted PSE-NHEKs developed mostly in aged mice. We now assume that they probably developed only after the aged microenvironment was established. Moreover, the skin hyperplasias and carcinomas did not develop at the site of the graft, but were highly disseminated, in accordance with very early acquisition of a migratory capacity by PSE-NHEKs. It is noteworthy that *in vitro*, PSE-NHEKs appear more sensitive to the pro-EMT effect of the senescent fibroblast secretome when the NHEKs are exposed to this secretome from the beginning of the culture. This suggests that the senescent microenvironment may contribute to the early transformation of keratinocytes.

We show in this study that the secretome of dermal fibroblasts is similar to that described for other cell models [33], [51]. Among the cytokines and growth factors of this secretome known to have pro-tumoral effects, IL-1, -6, and -8 appear to contribute very little or only indirectly to keratinocyte migration *in vitro* [52], [53], [54]. We therefore did not privilege them as potential inducers of the pro-EMT effect on PSE-NHEKs. HGF/SF has been implicated in keratinocyte migration during wound healing [55], [56], and its receptor, c-Met is essential to epithelial cell scattering [57]. Although the synthesis and secretion of HGF/SF are enhanced in senescent NHDFs, this factor can induce PSE-NHEK migration only very modestly, in agreement with the loss of C-Met in these cells. This, by the way, supports the view that PSE-NHEKs have undergone an EMT, since c-Met is normally expressed only by epithelial cells [58], [59]. The TGF-β1 pathway is known to be involved in EMT induction [35], partly through induction of TWIST-1 synthesis [60]. Senescent NHDFs show increased levels of the latent and active forms of TGF-β1, and although PSE-NHEKs show the same level TGF-β as young NHEKs, recombinant TGF-β1 failed to induce PSE-NHEK migration directly. It thus seems that neither HGF/SF nor TGF-β is responsible for the migration-promoting effect of SF-CM on PSE-NHEKs.

MMPs are also an established component of the senescent fibroblast secretome. Here we confirm this with with NHDFs, particularly as regards MMP-1 and MMP-2. Our results of in-gel and *in situ* zymography demonstrate the activity of the secreted MMPs, associated with reduced TIMP-1 expression. MMPs are widely known to promote late-phase cancer cell invasion through their proteinolytic action on extracellular matrix components. Their involvement in earlier phases of cancer initiation [61] and their ability to trigger EMT have also been demonstrated [62]. As components of the SASP, MMPs have been linked to EMT enhancement in pre-malignant or malignant epithelial cells [33]. Here we additionally find that MMP-1 and MMP-2 can stimulate migration of very early transformed cells having just evaded senescence. As none of the assays performed in our study involved an extracellular matrix, we speculate that MMPs must act directly on the biology of PSE-NHEKs through a cell surface receptor. This receptor must be activatable by proteolysis, since GM6001, an inhibitor of MMP proteinolytic activity, abolishes stimulation of PSE-NHEK migration by the senescent fibroblast secretome. PAR-1 appeared as a good candidate receptor because (i) it is oncogenic in NIH3T3 cells and its activation in various cancer cells has been linked to migration, invasion, and metastasis [43], [63], [64], [65], [66] [67], (ii) MMP-1 and MMP-2 secreted by skin fibroblasts have previously been linked to PAR-1-mediated migration of highly invasive breast cancer cells [43], (iii) and the PAR-1 gene is overexpressed in mammary cancer cell lines selected for strong EMT characters [68]. In control cultures and in contrast to c-met and TGF-βRII transcripts, the PAR-1 transcript level is higher in PSE-NHEKs than in young NHEKs. In PSE-NHEKs exposed to the senescent fibroblast secretome, it undergoes a further two-fold increase. This supports the view that the latter cells are transformed, as PAR-1 is known to be highly expressed in many cancer cells [43], [68], [69], [70], [71]. It is also known to be targeted to early endosomes and further processed for degradation to late endosomes and lysosomes [70]. We demonstrate here the involvement of the MMP-PAR-1 axis in senescent-fibroblast-secretome-promoted PSE-NHEK migration, in assays using recombinant MMPs or the PAR-1 agonist peptide SFLLRN, and in assays where either PAR-1 or its ligand MMP-1 or MMP-2 was functionally knocked down with siRNAs or blocking antibodies. Boire *et al.* have shown that MMP-1 can interact directly with PAR-1 and that it activates PAR-1 more efficiently than MMP-2 in breast cancer cells [43]. In our assays, the two MMPs showed an equal capacity, similar to that of SFLLRN, to reproduce the migration-promoting effect of SF-CM on PSE-NHEKs. It should be noted that MMP-2 is present in higher amount than MMP-1 in the senescent fibroblast secretome, suggesting that it might play a dominant role in promoting PSE-NHEK migration. Although PSE-NHEK/FGM, PSE-NHEK/YF-CM, and PSE-NHEK/SF-CM cells all display the same level of PAR-1, PSE-NHEK/SF-CM cells clearly respond more strongly to SF-CM than the others. The molecular basis for this enhanced response remains to be explored. We speculate that long-term culture with SF-CM results in the presence of PAR-1 co-activators and/or MMP co-receptors. As PAR-1 is not expressed *in vitro* in exponentially growing keratinocytes, we viewed it as a potential specific marker of PSE-NHEKs and used it in immunohistochemistry experiments on skin sections. Very interestingly, we detected PAR-1 only in rare, small subsets of keratinocytes in skin sections from aged donors or dysplastic samples. These few PAR-1-positive keratinocytes were found either in anarchic islets in the basal layer of the epidermis, or scattered through the underlying papillary dermis. This pattern of PAR-1 expression in skin suggests (i) that PSNE might be more than an *in vitro* artefact, developing *in vivo* in aged organisms; (ii) that the first post-senescence neoplastic cells might migrate very early. This last suggestion is strengthened by the fact that TWIST-1-positive cells were found scattered though the papillary dermis only in aged skins where PAR-1-positive cells were also scattered through this compartment. It has previously been shown that PAR-1 can govern cell shape and motility [41] and EMT-related migration [72], [73] and that transformed cells undergoing EMT can arise from hyperplasias and disseminate [74], [75], [76]. Here we show that EMT might take place even earlier, almost concomitantly with generation of the first neoplastic cells through senescence evasion. We also show that MMP activity is higher in the dermal compartment of aged skin samples than in that of young ones, particularly at the interface between the dermal and epidermal compartments. This suggests that migration of PAR-1-expressing keratinocytes might be directly stimulated by MMPs secreted by the underlying aged dermis.

In summary, we demonstrate that a MMP-PAR-1 axis may be involved in the very early steps of non-melanoma skin carcinogenesis. It would be set up with aging, through coordinated modifications of both senescing fibroblasts of the dermis and keratinocytes initiating neoplastic transformation through senescence evasion. Since a diagnosis of non-melanoma skin cancer is associated with an increased risk of developing other primary carcinomas [20], the present mechanism could be relevant to other age-associated carcinomas.

## Supporting Information

Figure S1 Typical senescence of NHDFs *in vitro*. **A**. Growth curve of NHDFs from donor 2F1966. The young fibroblasts used in this study were taken at 10–20 PDs. Senescent fibroblasts were taken after day 200. This growth curve is representative of more than 30 experiments performed with cells from this donor and is also representative of growth curves obtained with cells from other donors. **B**. Morphologies of young and senescent NHDFs. Senescent NHDFs display the typical enlarged and flattened morphology associated with vesicle accumulation. **C**. SA-β-Gal assay. The percentage of SA-β-Gal-positive cells is given.
(TIF)

Figure S2 Complementary results, statistics, and quantification of the results of Figure 1. **A.** Statistical analysis of the results in Figure 1B. **B.** Western-blot analysis of E-cadherin and vimentin in young NHEKs cultured in the presence of the different conditioned media that later have given rise to the PSE-NHEKs assayed in Figure 1C. In contrast to the results obtained with PSE-NHEKs, vimentin and the 80-kDa and 120-kDa forms of E-cadherin were not affected by the conditioned media. Similar results were obtained with a second cell lysate. **C.** Densitometric analysis of the western blots of Figure S2B and Figure 1C. The density of each band was divided by that of the corresponding GAPDH band and the obtained value was normalized with respect to the control value obtained in non-supplemented KGM. **D**. Statistical analysis of the results of Figure 1D.
(TIF)

Figure S3 Expression of growth factors and cytokines in senescent dermal fibroblasts. RT-qPCR analysis of: **A.** growth factor transcripts (TGF-β1, HGF-SF, VEGF, AREG and EGF) and **B**. transcripts (MCP-1, IL-6, IL-8, Gro-1 and SDF-1) in young and senescent NHDFs (donor 2F1966). Results are means of triplicates±SD (**: p<0.01). Data are representative of 4 independent experiments performed with 2 different donors.
(TIF)

Figure S4 Neither the HGF-SF/c-Met nor the TGF-β1/TGF-bRII axis is involved in SF-CM-induced migration of PSE-NHEKs. **A.** Western-blot analysis of HGF/SF in YF-CM and SF-CM (media conditioned by NHDFs obtained from donor 2F1966). **B.** ELISA analysis of the HGF/SF concentration in the same conditioned media. Data are representative of 3 independent experiments performed with 2 different donors. **C.** Migration assays. Young NHEKs and PSE-NHEKs cultured in control KGM were starved in fresh KBM and seeded in KBM onto the tops of Transwell® chambers for migration assays. Recombinant HGF-SF (10 ng/ml in KBM) was used as attractant. After a 30-h incubation at 37°C, cells having migrated were counted in ten random fields per well. Results are given as means±SD of triplicates (**: p<0.01). Data are representative of 3 independent experiments performed with 2 different donors. **D.** PSE-NHEK/SF-CM were used in migration assays as in (C). FGM, SF-CM, or FGM supplemented with recombinant TGF-β1 (10 ng/ml) or HGF/SF (10 ng/ml), was used as attractant. Results are means ±SD of triplicates (**: p<0.01). Data are representative of 2 independent experiments. **E.** RT-qPCR analysis of c-met transcripts in young NHEKs and PSE-NHEKs (donor 2F1958) cultured under the different conditions described in Figure 1A (KGM, or 10% FGM, YF-CM, or SF-CM with 90% KGM). Results are means ±SD of triplicates (**: p<0.01). Data are representative of 3 independent experiments. F. Western-blot analyses of the c-Met protein in young- or PSE-NHEKs from the different culture conditions. GAPDH was used as loading control. G. RT-qPCR analysis of the TGF-β1 receptor TGF-β RII transcripts in young NHEKs and PSE-NHEKs (donor 2F1958) cultured under the different conditions described in figure 1A (KGM, or 10% FGM, YF-CM, or SF-CM with 90% KGM). Results are means of triplicates ± SD. None of the differences between KGM and the other culture conditions is statistically significant. Data are representative of 3 independent experiments. **H.** Western blot analysis of TGF-β1 latent and active forms in lysates of young and senescent NHDFs (donor 2F1966). GAPDH was used as loading control. Data are representative of 3 independent experiments performed with cells from 2 different donors.
(TIF)

Figure S5 Supporting information for Figure 2. **A.** Densitometric analysis of the western blots of Figure 2B. The density of each band was divided by that of the corresponding actin band, and the obtained value was divided by the value obtained for young NHDFs. **B.** Western-blot analysis of MMP-1 in concentrated conditioned media from cultures of young (YF) and senescent (SF) NHDFs. Actin was used as loading control. Data are representative of two independent experiments **C**. Concentrated conditioned medium from a culture of young (YF-CM) or senescent fibroblasts (SF-CM) was resolved by electrophoresis in a silver-stained preparative gel. A band corresponding to the molecular weight of TIMP-1 was almost absent in SF-CM. The band corresponding to the molecular weight of albumin was not significantly affected. **D**. Identification of the presumed TIMP-1 by mass spectrometry. The bands believed to contain TIMP-1 were manually excised, washed with ultrapure water until totally destained, and then dried after adding 100 µL ACN and incubating for 10 min. After the supernatant was discarded, the tubes were left open for 10 min to allow complete solvent evaporation. Spots were rehydrated with a solution containing Trypsin Enhancer (Promega) in 50 mM ammonium bicarbonate and a solution containing 3 µL of 40 µg/mL Trypsin Gaged (Promega) in 50 mM acetic acid and then trypsin-digested. Peptide extraction was carried out in two steps according to the manufacturer’s protocol. MALDI-TOF MS was then performed with a Voyager DE STR mass spectrometer (PerSeptive Biosystems, Framingham, MA) equipped with a 337.1-nm nitrogen laser and a delayed extraction device (125 msec). Protein identification was performed by peptide mass fingerprinting, conducted by running the MASCOT web searcher (http://www.matrixscience.com/, Matrix Science, UK) against NCBInr 20100312 (10570301 sequences; 3602205473 residues).
(TIF)

Figure S6 Checking of the efficacy of MMP-1 and MMP-2 inhibition/knockdown in senescent NHDFs and of PAR-1 inhibition/knockdown in PSE-NHEKs. **A.** In-gel zymography performed to test the ability of the broad-spectrum MMP inhibitor GM6001 to inhibit the proteinolytic activity of MMP-2 present in serum (FGM) or in the media conditioned by young (YF-CM) or senescent (SF-CM) NHDFs. The analysis was done by SDS-PAGE with a gel containing 0.1% gelatin. White bands denote the presence of proteins with gelatinolytic activity. GM6001 was added at 12.5 µM final concentration to the three different media. As a control the same volume of DMSO (the diluent used for GM6001) was added to the same media. **B.** In-gel zymography performed to test the ability of siRNAs to knock down MMP-1 and MMP-2 expression. Senescent NHDFs (donor 2F1966) were transfected with a pool of 4 non-targeting control siRNAs or a pool of 4 MMP-1 or MMP-2 siRNAs (20 µM, Dharmacon). After 72 h, the corresponding conditioned media were collected and used for gelatin zymography as in A. KGM conditioned by untransfected senescent fibroblasts (Mock) and FGM were loaded as controls. Results are representative of two independent experiments. **C.** Western-blot analysis of the efficacy of siRNAs against PAR-1. PSE-NHEKs (donor 2F1958) cultured with FGM, YF-CM, or SF-CM were transfected with a pool of 4 non-targeting control siRNAs (si Ctrl) or a pool of 4 PAR-1 siRNAs (si PAR-1) (20 µM, Dharmacon). After 72 h, the cells were starved in KBM for 16 h to replenish the pool of PAR-1. Then the cells were lysed in Laemmli loading buffer and used for western blotting. Results are representative of two independent experiments. **D**. Migration assays. PSE-NHEK/FGM and PSE-NHEK/YF-CM cells (donor 2F1958) were transfected with siRNAs as in C. After 72 h, the cells were starved in KBM for 16 h, trypsinized, and seeded in KBM into Transwell® plates. YF-CM or SF-CM was used as attractant. The experiment was also done in parallel with PSE-NHEK/SF-CM (results shown in Figure 4F). Cells were left in the Transwell® chamber for 30 h at 37°C, after which cells having migrated were counted in ten random fields per well, each condition being tested in triplicate. Results are given as means±SD (**: p<0.01). Similar results were obtained in three independent experiments.
(TIF)

Figure S7 Quantitative analyses of the western blotting experiments of Figures 4 and 5. **A**. Densitometric analysis of the western blots of Figure 4B, lower panel (**A**), 4C (**B**), 5A (**C**), 5B (**D**) 5C (**E**). The optical density of each band was divided by that of the corresponding GAPDH band, and the obtained value was divided by that obtained for the control.
(TIF)

Figure S8 PAR-1 activation by CM components is monitored by its internalization and accumulation in bafilomycin-sensitive vesicles. PSE-NHEK/YF-CM cells (upper panels) and PSE-NHEK/SF-CM cells (lower panels) were seeded onto coverglasses, starved for 16 h in KBM to allow PAR-1 re-expression at the membrane (from active transcription), and simultaneously treated with bafilomycin A1 (5 nM; *Streptomyces griseus* B 1793), or its solvent DMSO. The PSE-NHEK/YF-CM and PSE-NHEK/SF-CM cells were then re-stimulated or not for 1 h with either YF-CM or SF-CM, respectively. Finally, PAR-1 immunostaining was carried out as described under Materials and Methods. Scale bar: 10 µm. Pictures are representative of 2 independent experiments.
(TIF)

Table S1 List of primers used in qRT-PCR experiments.
(DOC)

Table S2 Characteristics of skin sample donors and presence/absence of TWIST-1, PAR-1, and MMPs in the corresponding biopsies.
(DOC)
