# Supplementary figures and images for: Senescent Fibroblasts Enhance Early Skin Carcinogenic Events via a Paracrine MMP-PAR-1 Axis
Source: PLoS One. 2013 May 10;8(5):e63607. doi: 10.1371/journal.pone.0063607 (PMC3651095; doi:10.1371/journal.pone.0063607)

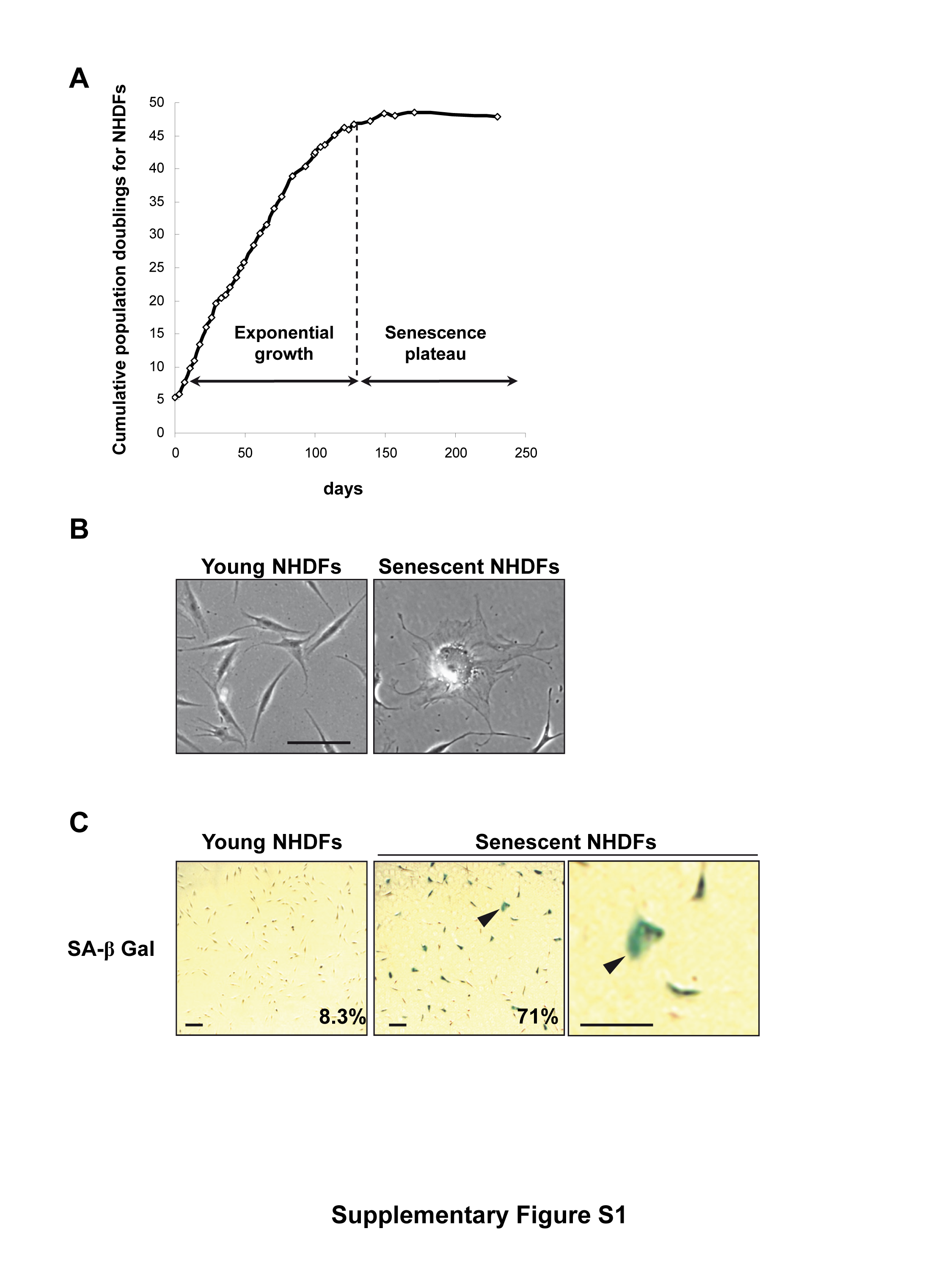

Supplement: Figure S1 — Typical senescence of NHDFs in vitro. A. Growth curve of NHDFs from donor 2F1966. The young fibroblasts used in this study were taken at 10–20 PDs. Senescent fibroblasts were taken after day 200. This growth curve is representative of more than 30 experiments performed with cells from this donor and is also representative of growth curves obtained with cells from other donors. B. Morphologies of young and senescent NHDFs. Senescent NHDFs display the typical enlarged and flattened morphology associated with vesicle accumulation. C. SA-β-Gal assay. The percentage of SA-β-Gal-positive cells is given. (TIF) [file pone.0063607.s001.tif]

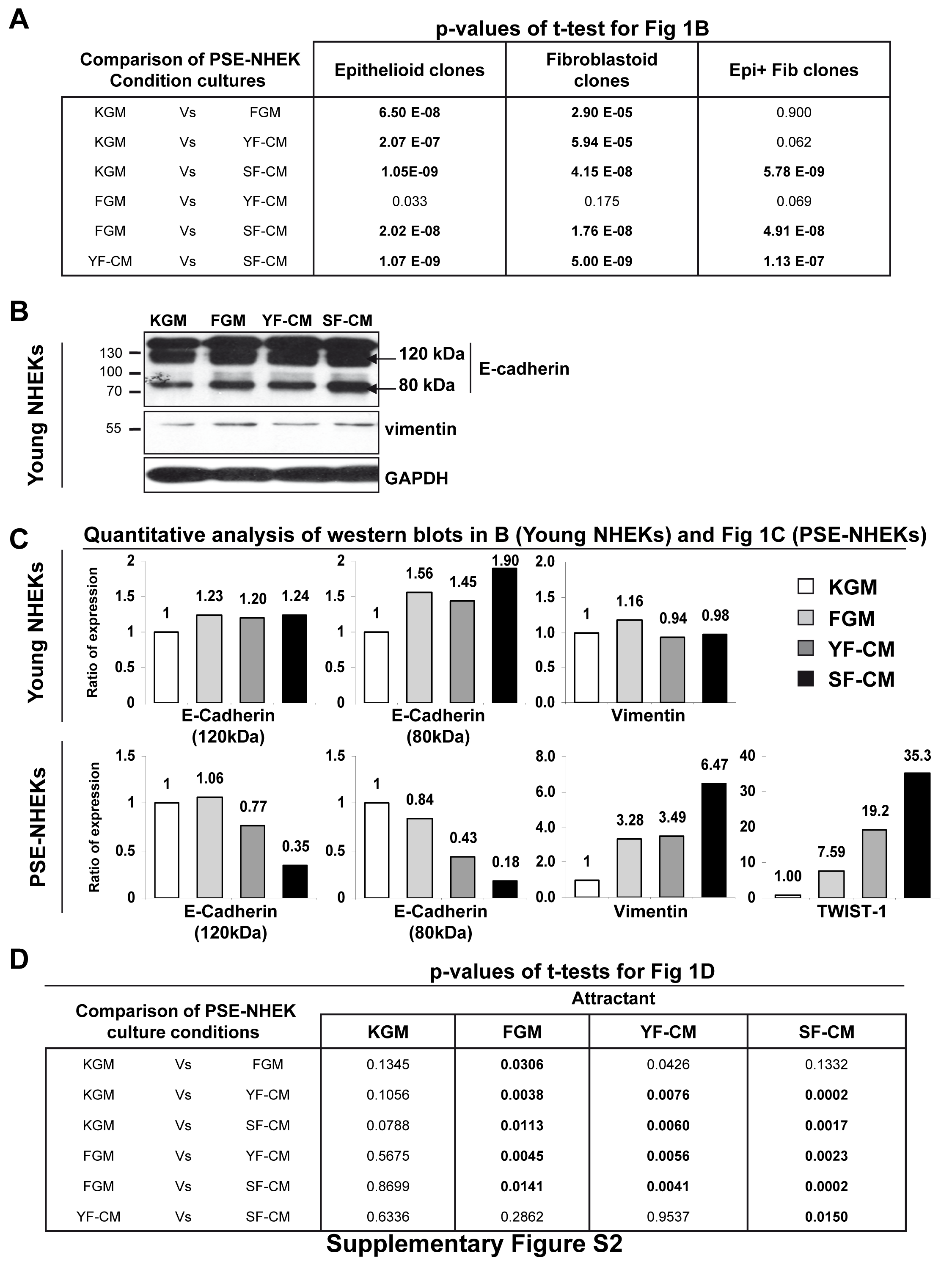

Supplement: Figure S2 — Complementary results, statistics, and quantification of the results of Figure 1. A. Statistical analysis of the results in Figure 1B. B. Western-blot analysis of E-cadherin and vimentin in young NHEKs cultured in the presence of the different conditioned media that later have given rise to the PSE-NHEKs assayed in Figure 1C. In contrast to the results obtained with PSE-NHEKs, vimentin and the 80-kDa and 120-kDa forms of E-cadherin were not affected by the conditioned media. Similar results were obtained with a second cell lysate. C. Densitometric analysis of the western blots of Figure S2B and Figure 1C. The density of each band was divided by that of the corresponding GAPDH band and the obtained value was normalized with respect to the control value obtained in non-supplemented KGM. D. Statistical analysis of the results of Figure 1D. (TIF) [file pone.0063607.s002.tif]

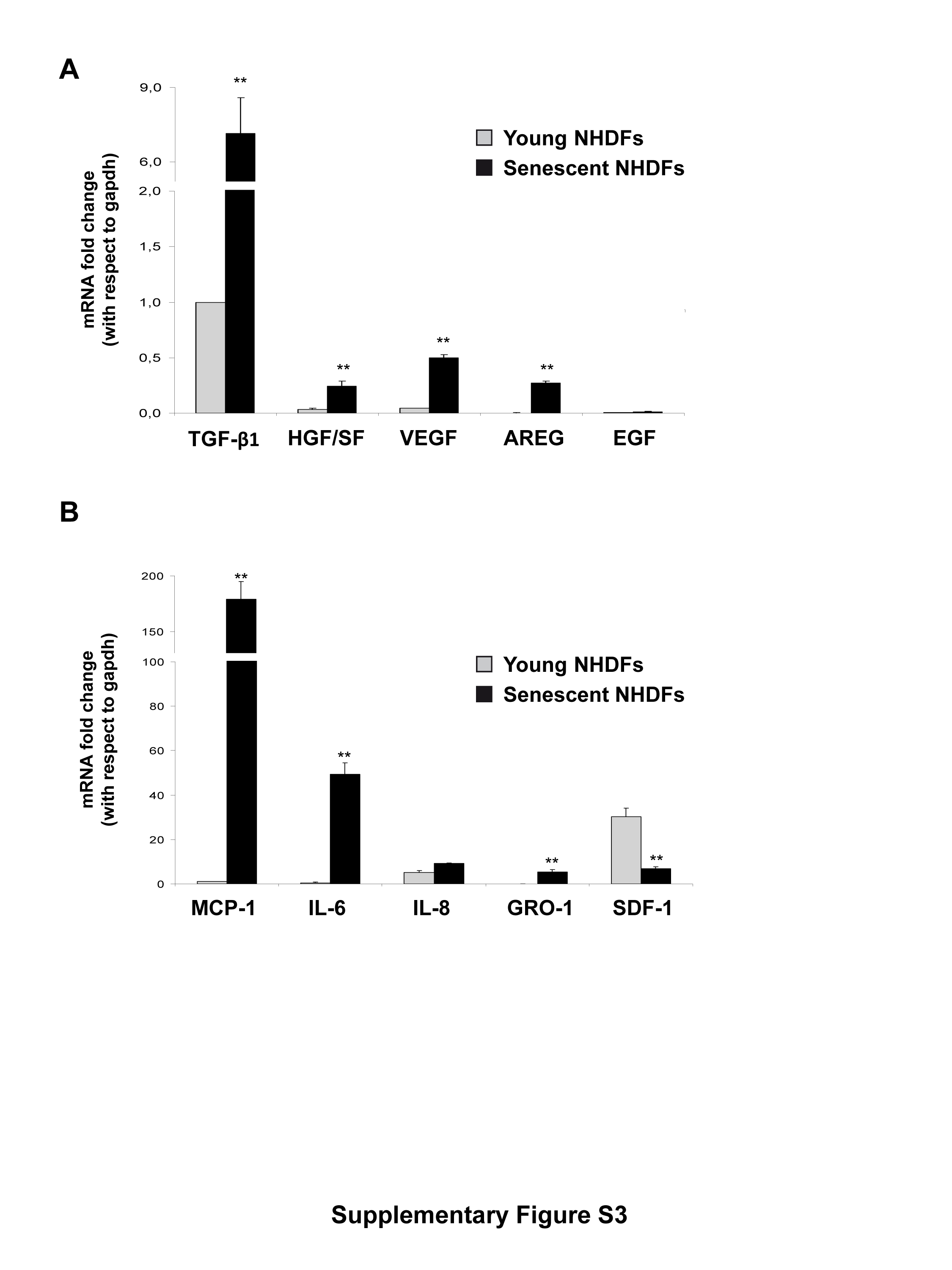

Supplement: Figure S3 — Expression of growth factors and cytokines in senescent dermal fibroblasts. RT-qPCR analysis of: A. growth factor transcripts (TGF-β1, HGF-SF, VEGF, AREG and EGF) and B. transcripts (MCP-1, IL-6, IL-8, Gro-1 and SDF-1) in young and senescent NHDFs (donor 2F1966). Results are means of triplicates±SD (**: p<0.01). Data are representative of 4 independent experiments performed with 2 different donors. (TIF) [file pone.0063607.s003.tif]

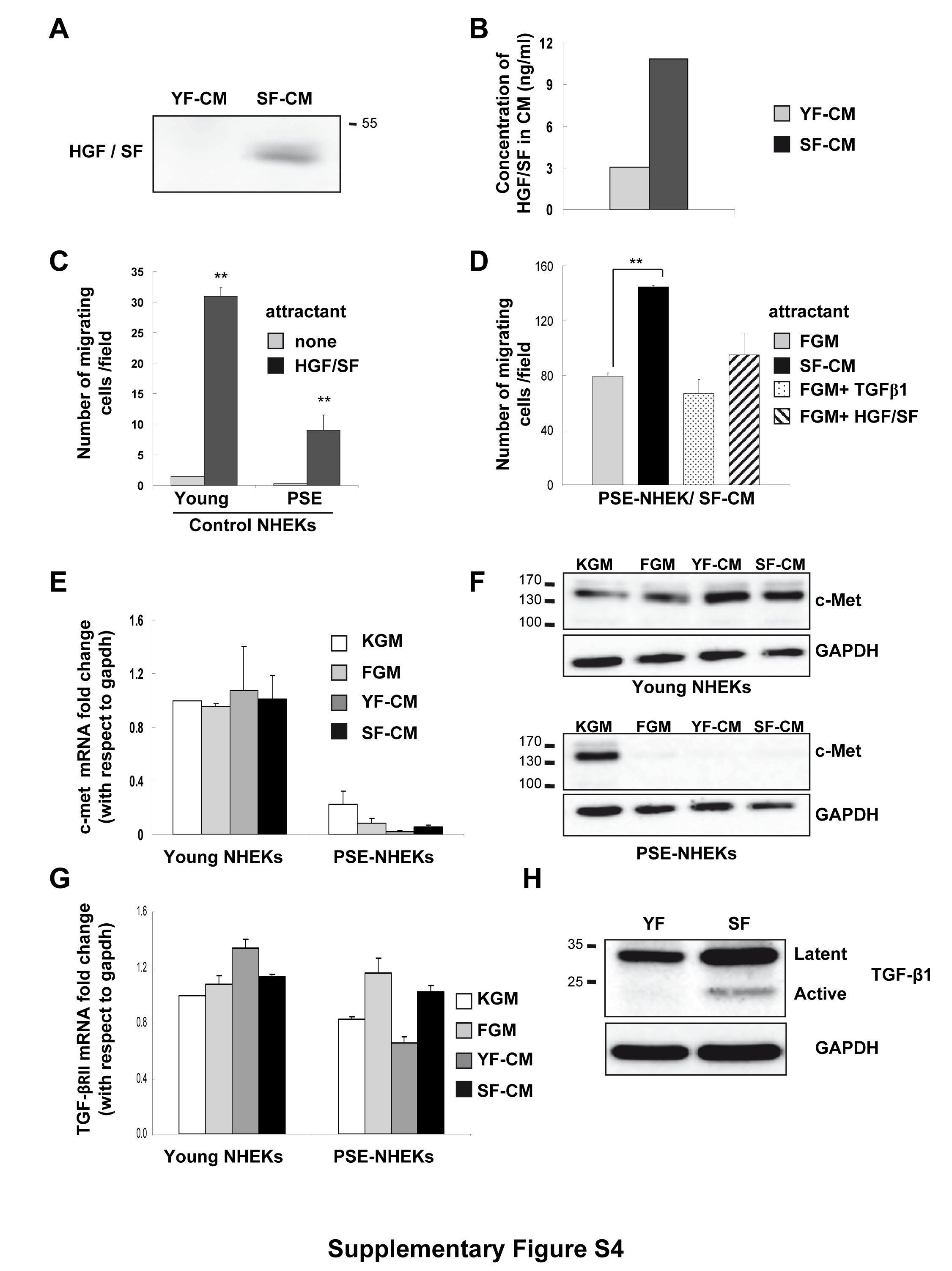

Supplement: Figure S4 — Neither the HGF-SF/c-Met nor the TGF-β1/TGF-bRII axis is involved in SF-CM-induced migration of PSE-NHEKs. A. Western-blot analysis of HGF/SF in YF-CM and SF-CM (media conditioned by NHDFs obtained from donor 2F1966). B. ELISA analysis of the HGF/SF concentration in the same conditioned media. Data are representative of 3 independent experiments performed with 2 different donors. C. Migration assays. Young NHEKs and PSE-NHEKs cultured in control KGM were starved in fresh KBM and seeded in KBM onto the tops of Transwell® chambers for migration assays. Recombinant HGF-SF (10 ng/ml in KBM) was used as attractant. After a 30-h incubation at 37°C, cells having migrated were counted in ten random fields per well. Results are given as means±SD of triplicates (**: p<0.01). Data are representative of 3 independent experiments performed with 2 different donors. D. PSE-NHEK/SF-CM were used in migration assays as in (C). FGM, SF-CM, or FGM supplemented with recombinant TGF-β1 (10 ng/ml) or HGF/SF (10 ng/ml), was used as attractant. Results are means ±SD of triplicates (**: p<0.01). Data are representative of 2 independent experiments. E. RT-qPCR analysis of c-met transcripts in young NHEKs and PSE-NHEKs (donor 2F1958) cultured under the different conditions described in Figure 1A (KGM, or 10% FGM, YF-CM, or SF-CM with 90% KGM). Results are means ±SD of triplicates (**: p<0.01). Data are representative of 3 independent experiments. F. Western-blot analyses of the c-Met protein in young- or PSE-NHEKs from the different culture conditions. GAPDH was used as loading control. G. RT-qPCR analysis of the TGF-β1 receptor TGF-β RII transcripts in young NHEKs and PSE-NHEKs (donor 2F1958) cultured under the different conditions described in figure 1A (KGM, or 10% FGM, YF-CM, or SF-CM with 90% KGM). Results are means of triplicates ± SD. None of the differences between KGM and the other culture conditions is statistically significant. Data are representative of 3 independent experiments. H [file pone.0063607.s004.tif]

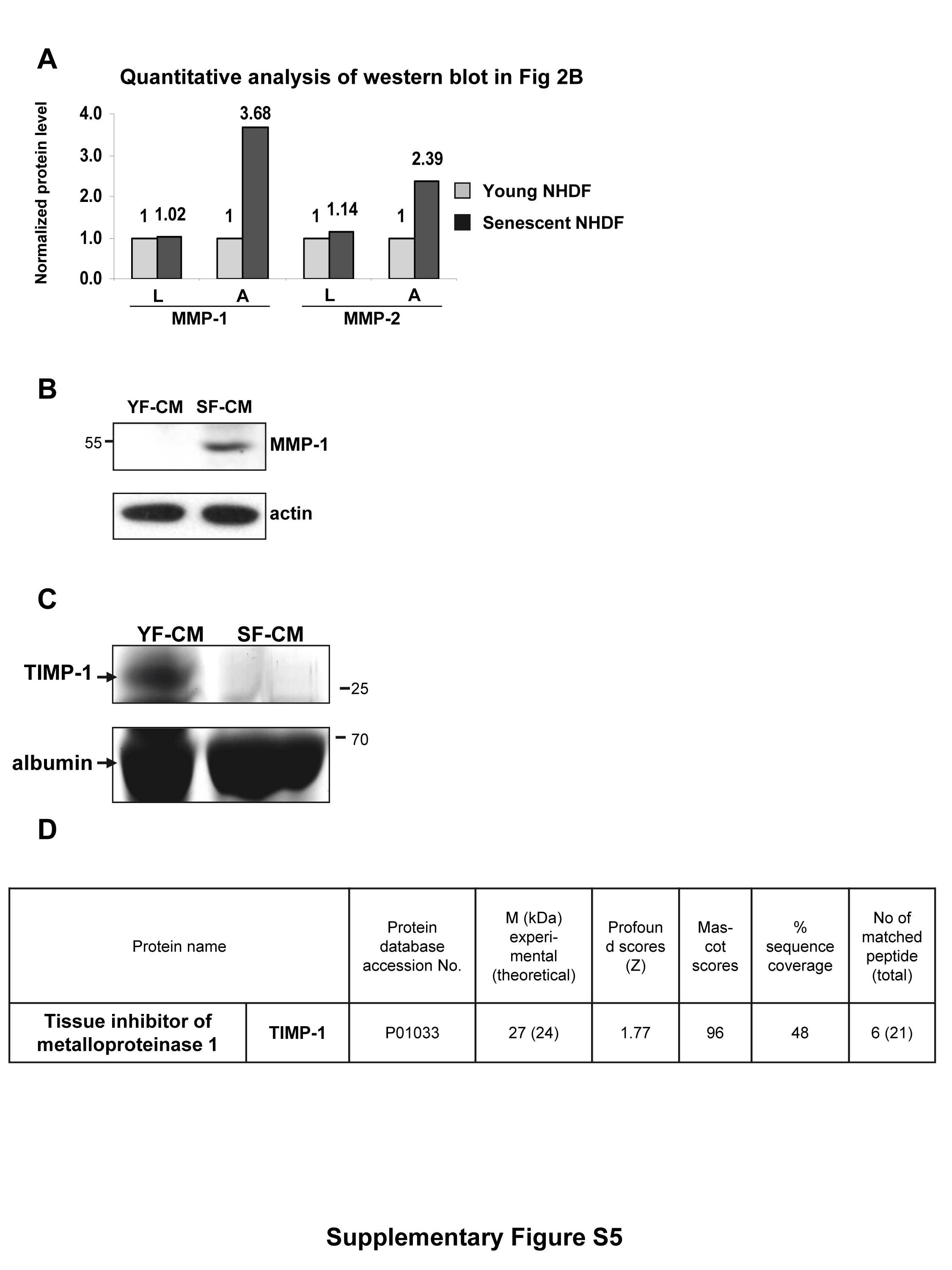

Supplement: Figure S5 — Supporting information for Figure 2. A. Densitometric analysis of the western blots of Figure 2B. The density of each band was divided by that of the corresponding actin band, and the obtained value was divided by the value obtained for young NHDFs. B. Western-blot analysis of MMP-1 in concentrated conditioned media from cultures of young (YF) and senescent (SF) NHDFs. Actin was used as loading control. Data are representative of two independent experiments C. Concentrated conditioned medium from a culture of young (YF-CM) or senescent fibroblasts (SF-CM) was resolved by electrophoresis in a silver-stained preparative gel. A band corresponding to the molecular weight of TIMP-1 was almost absent in SF-CM. The band corresponding to the molecular weight of albumin was not significantly affected. D. Identification of the presumed TIMP-1 by mass spectrometry. The bands believed to contain TIMP-1 were manually excised, washed with ultrapure water until totally destained, and then dried after adding 100 µL ACN and incubating for 10 min. After the supernatant was discarded, the tubes were left open for 10 min to allow complete solvent evaporation. Spots were rehydrated with a solution containing Trypsin Enhancer (Promega) in 50 mM ammonium bicarbonate and a solution containing 3 µL of 40 µg/mL Trypsin Gaged (Promega) in 50 mM acetic acid and then trypsin-digested. Peptide extraction was carried out in two steps according to the manufacturer’s protocol. MALDI-TOF MS was then performed with a Voyager DE STR mass spectrometer (PerSeptive Biosystems, Framingham, MA) equipped with a 337.1-nm nitrogen laser and a delayed extraction device (125 msec). Protein identification was performed by peptide mass fingerprinting, conducted by running the MASCOT web searcher (http://www.matrixscience.com/, Matrix Science, UK) against NCBInr 20100312 (10570301 sequences; 3602205473 residues). (TIF) [file pone.0063607.s005.tif]

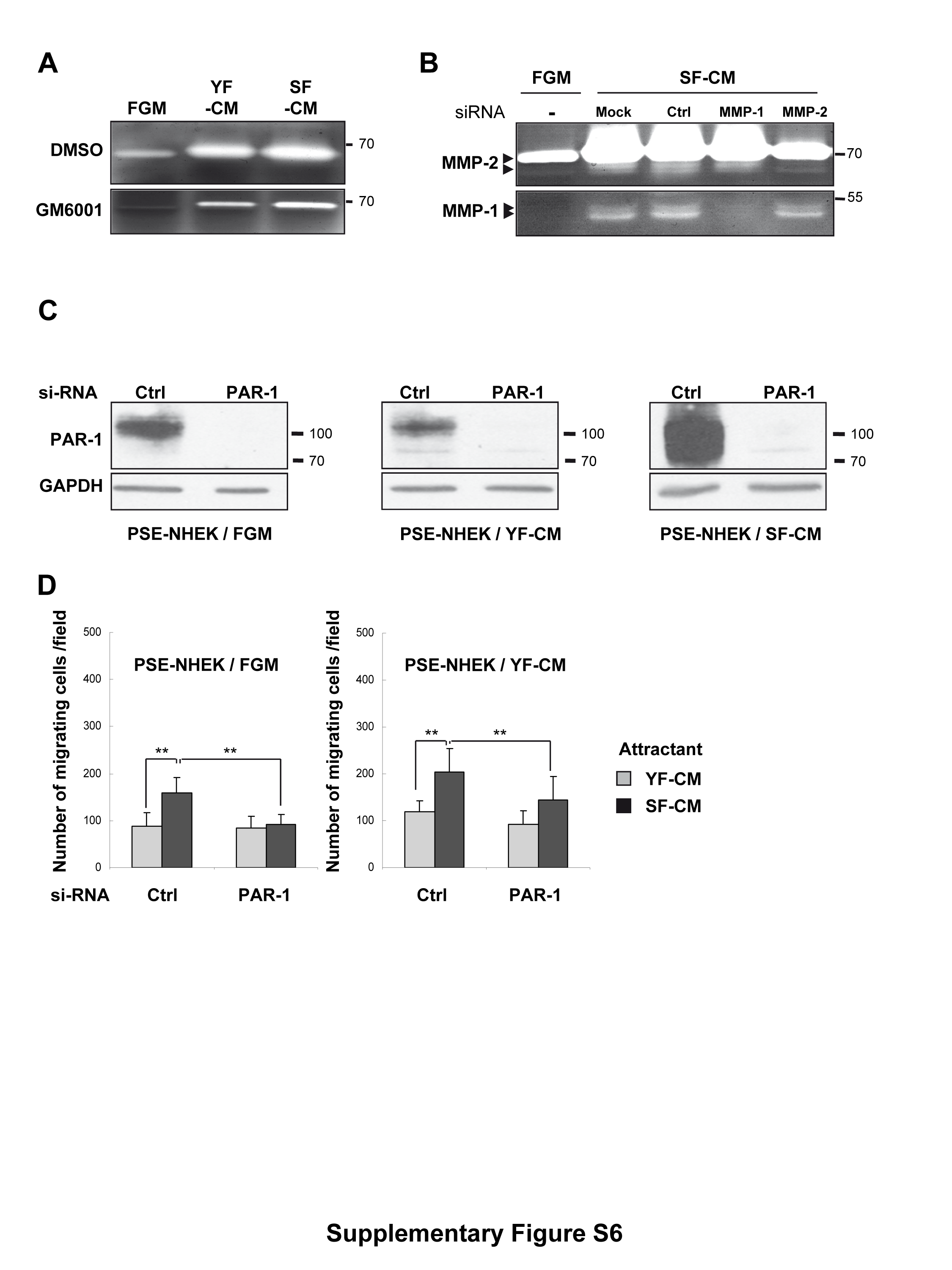

Supplement: Figure S6 — Checking of the efficacy of MMP-1 and MMP-2 inhibition/knockdown in senescent NHDFs and of PAR-1 inhibition/knockdown in PSE-NHEKs. A. In-gel zymography performed to test the ability of the broad-spectrum MMP inhibitor GM6001 to inhibit the proteinolytic activity of MMP-2 present in serum (FGM) or in the media conditioned by young (YF-CM) or senescent (SF-CM) NHDFs. The analysis was done by SDS-PAGE with a gel containing 0.1% gelatin. White bands denote the presence of proteins with gelatinolytic activity. GM6001 was added at 12.5 µM final concentration to the three different media. As a control the same volume of DMSO (the diluent used for GM6001) was added to the same media. B. In-gel zymography performed to test the ability of siRNAs to knock down MMP-1 and MMP-2 expression. Senescent NHDFs (donor 2F1966) were transfected with a pool of 4 non-targeting control siRNAs or a pool of 4 MMP-1 or MMP-2 siRNAs (20 µM, Dharmacon). After 72 h, the corresponding conditioned media were collected and used for gelatin zymography as in A. KGM conditioned by untransfected senescent fibroblasts (Mock) and FGM were loaded as controls. Results are representative of two independent experiments. C. Western-blot analysis of the efficacy of siRNAs against PAR-1. PSE-NHEKs (donor 2F1958) cultured with FGM, YF-CM, or SF-CM were transfected with a pool of 4 non-targeting control siRNAs (si Ctrl) or a pool of 4 PAR-1 siRNAs (si PAR-1) (20 µM, Dharmacon). After 72 h, the cells were starved in KBM for 16 h to replenish the pool of PAR-1. Then the cells were lysed in Laemmli loading buffer and used for western blotting. Results are representative of two independent experiments. D. Migration assays. PSE-NHEK/FGM and PSE-NHEK/YF-CM cells (donor 2F1958) were transfected with siRNAs as in C. After 72 h, the cells were starved in KBM for 16 h, trypsinized, and seeded in KBM into Transwell® plates. YF-CM or SF-CM was used as attractant. The experiment was also done in parallel with PSE-NHEK/SF-CM [file pone.0063607.s006.tif]

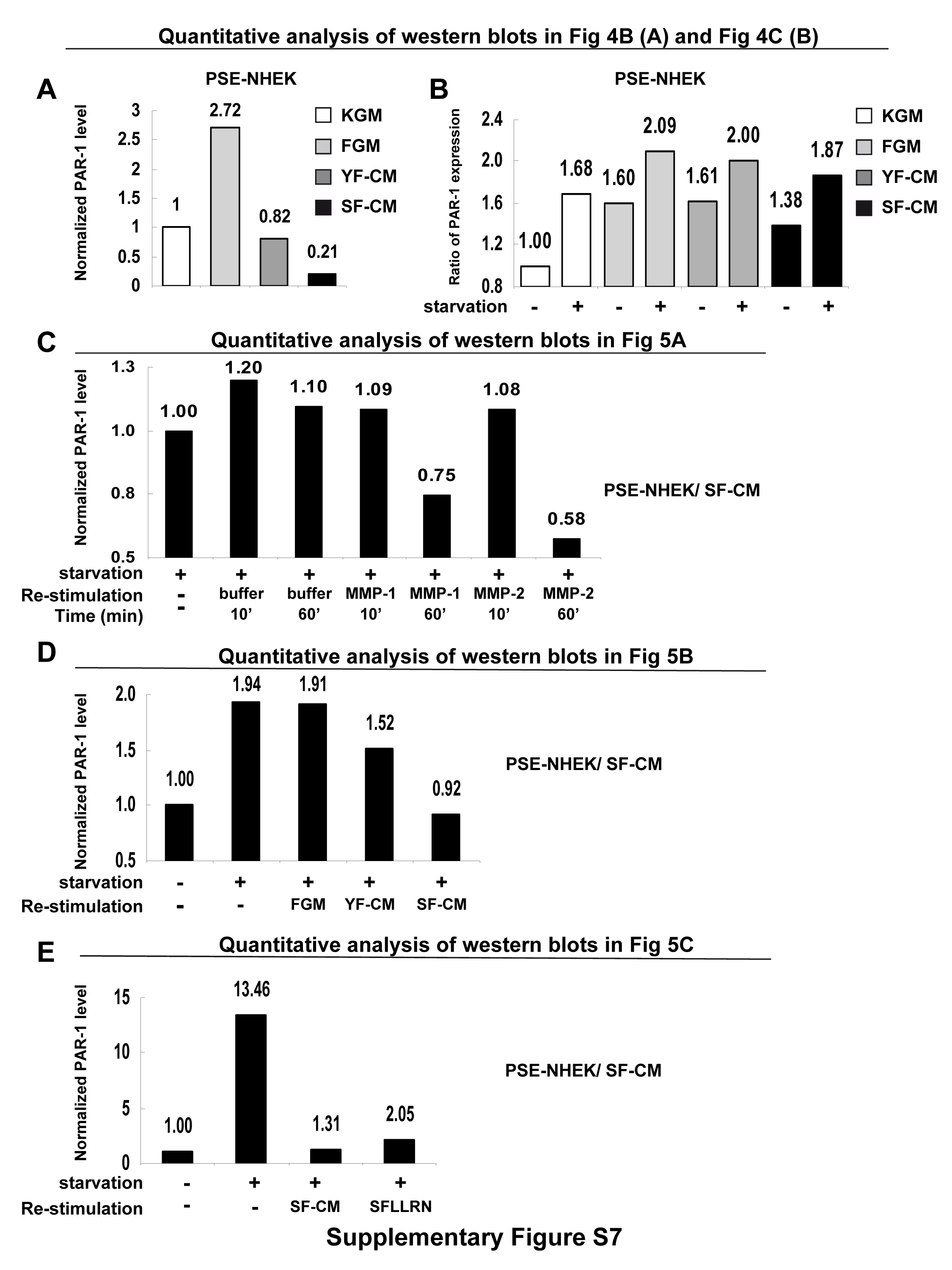

Supplement: Figure S7 — Quantitative analyses of the western blotting experiments of Figures 4 and 5. A. Densitometric analysis of the western blots of Figure 4B, lower panel (A), 4C (B), 5A (C), 5B (D) 5C (E). The optical density of each band was divided by that of the corresponding GAPDH band, and the obtained value was divided by that obtained for the control. (TIF) [file pone.0063607.s007.tif]

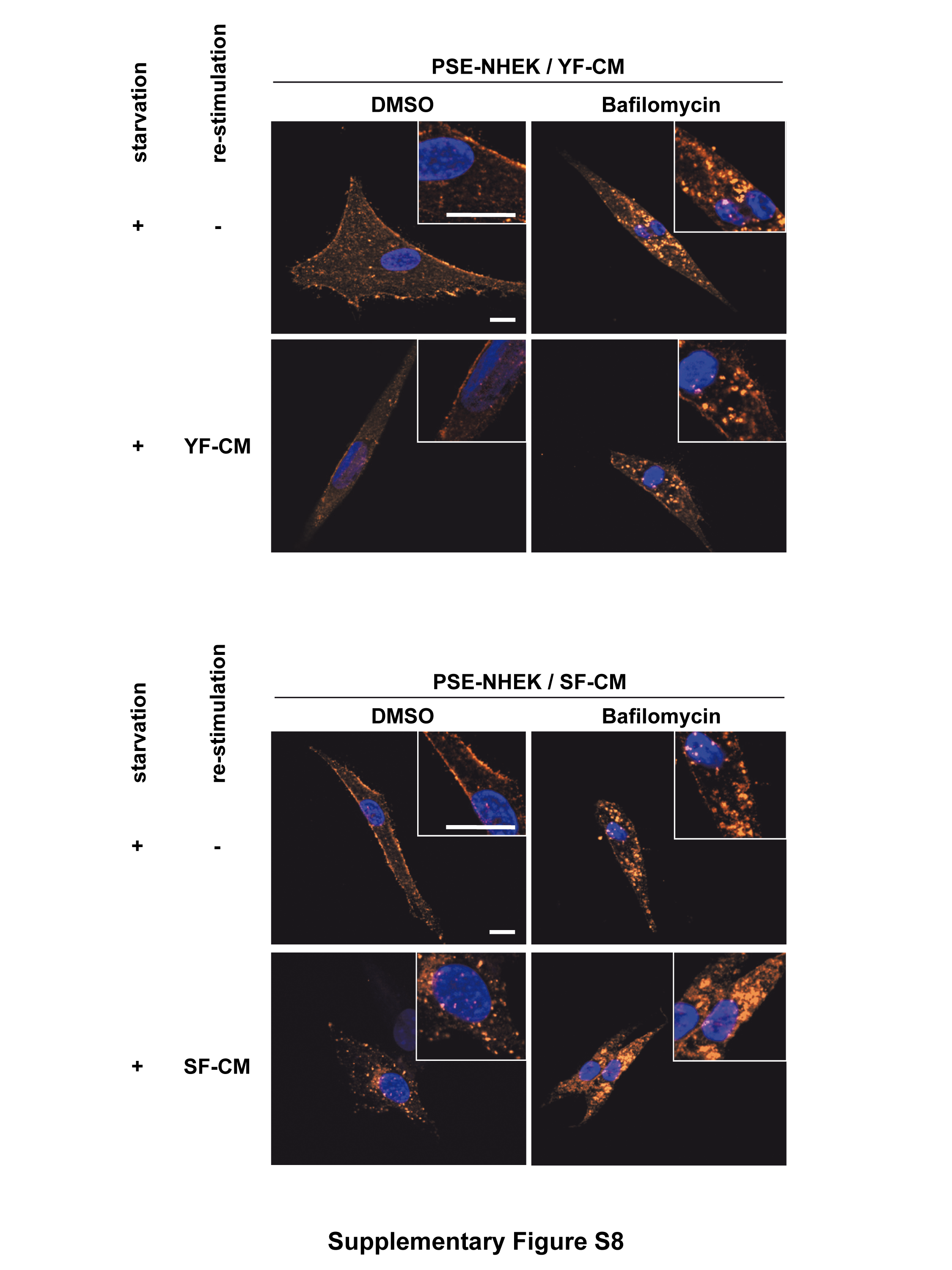

Supplement: Figure S8 — PAR-1 activation by CM components is monitored by its internalization and accumulation in bafilomycin-sensitive vesicles. PSE-NHEK/YF-CM cells (upper panels) and PSE-NHEK/SF-CM cells (lower panels) were seeded onto coverglasses, starved for 16 h in KBM to allow PAR-1 re-expression at the membrane (from active transcription), and simultaneously treated with bafilomycin A1 (5 nM; Streptomyces griseus B 1793), or its solvent DMSO. The PSE-NHEK/YF-CM and PSE-NHEK/SF-CM cells were then re-stimulated or not for 1 h with either YF-CM or SF-CM, respectively. Finally, PAR-1 immunostaining was carried out as described under Materials and Methods. Scale bar: 10 µm. Pictures are representative of 2 independent experiments. (TIF) [file pone.0063607.s008.tif]
