# Supplementary material for: Senescent Fibroblasts Enhance Early Skin Carcinogenic Events via a Paracrine MMP-PAR-1 Axis
Source: PLoS One. 2013 May 10;8(5):e63607. doi: 10.1371/journal.pone.0063607 (PMC3651095; doi:10.1371/journal.pone.0063607)
Supplement: Table S1 — List of primers used in qRT-PCR experiments. (DOC) [file pone.0063607.s009.doc]

**Supplementary Table S1: List of primers used in qRT-PCR experiments.**

|  | **Forward primer (5'->3')** | **Reverse primer (5'->3')** |
| --- | --- | --- |
| **MMP-1** | GAGCAAACACATCTGAGGTACAGGA | TTGTCCCGATGATCTCCCCTGACA |
| **MMP-2** | AGATCTTCTTCTTCAAGGACCGGTT | GGCTGGTCAGTGGCTTGGGGTA |
| **MMP-3** | GATCTCTTCATTTTGGCCATCTCTTC | CTCCAGTATTTGTCCTCTACAAAGAA |
| **MMP-9** | GCGCTGGGCTTAGATCATTCCTCA | GCAGCGCGGGCCACTTGTC |
| **MT1-MMP** | GGATACCCAATGCCCATTGGCCA | CCATTGGGCATCCAGAAGAGAGC |
| **PAR-1** | GTGAAGCGGAGCAGCCCGA | GGGCCGCACAGACTGAAGCA |
| **TGF-β1** | ACTACTACGCCAAGGAGGTCAC | ACGTGCTGCTCCACTTTTAACT |
| **HGF/SF** | TCCTGGTATTTTTGTCCGAG | AAACAAAACAACAGAAAACACC |
| **VEGF** | GAGCGGAGCCGCGAGAAGTG | CGCCTCACCCGTCCATGAGC |
| **AREG** | TCTTGATACTCGGCTCAGGCCA | TCCATCAGCACTGTGGTCCCC |
| **EGF** | TGACACTTGGGAGCCTGAGCA | AGGGCGTATCCCTCTGCACACA |
| **MCP-1** | CTCGCGAGCTATAGAAGAATCAC | TCAAAACATCCCAGGGGTAGAACT |
| **IL-6** | ATGTAGCCGCCCCACACA | CCAGTGCCTCTTTGCTGCTT |
| **IL-8** | TTGCCCAGAAGCGAACAGAC | CCAGTGGAGGCATAAGAGCAGA |
| **Gro-1** | CTGAGGAGCCTGCAACATGCCA | TGTGCACATACATTCCCCTGCC |
| **SDF-1** | ACCGCGCTCTGCCTC | CGTTGGCTCTGGCAACATG |
| **c-Met** | TTTCAAATGGCCACGGGACAACACA | TGGGCTGGGGTATAACATTCAAGA |
| **TGF-RII** | AGCAACTGCAGCATCACCTCC | GGGAGCTTGGGGTCATGGCA |
| **GAPDH** | ATCTCTGCCCCCTCTGCT | GCAGGAGGCATTGCTGAT |
