# Supplementary material for: Senescent Fibroblasts Enhance Early Skin Carcinogenic Events via a Paracrine MMP-PAR-1 Axis
Source: PLoS One. 2013 May 10;8(5):e63607. doi: 10.1371/journal.pone.0063607 (PMC3651095; doi:10.1371/journal.pone.0063607)
Supplement: Table S2 — Characteristics of skin sample donors and presence/absence of TWIST-1, PAR-1, and MMPs in the corresponding biopsies. (DOC) [file pone.0063607.s010.doc]

**Supplementary Table S2:** Characteristics of skin sample donors and presence/absence of TWIST-1, PAR-1, and MMPs in the corresponding biopsies, as established by immunohistodetection and zymography (ND: not determined, -: negative, +: positive).

| 1. **Skin biopsies from healthy “young” donors** | | | | | |
| --- | --- | --- | --- | --- | --- |
| **N° donor** | **Age** | **Sex** | **TWIST-1** | **PAR-1** | **MMPs** |
| 869/07 | 26 | female | **-** | **-** | ND |
| 455/05 | 29 | female | **-** | **-** | ND |
| 32645/09 | 34 | male | **-** | **-** | **-** |
| 33410/10 | 38 | female | **-** | **-** | **-** |
| 35968/10 | 38 | female | **+** | **-** | **-** |
| 1. **Skin biopsies from healthy “old” donors** | | | | | |
| **N° donor** | **Age** | **Sex** | **TWIST-1** | **PAR-1** | **MMPs** |
| 13317/10 | 60 | male | **-** | **-** | **+** |
| 9238/09 | 75 | male | **-** | **-** | ND |
| 15727/11 | 76 | male | **+** | **+** | ND |
| 21075/09 | 80 | male | **+** | **+** | **+** |
| 17608/08 | 80 | female | **+** | **-** | **+** |
| 38989/09 | 83 | male | **-** | **-** | **+** |
| 28010/09 | 89 | male | **+** | **+** | **+** |
| 1. **Skin biopsies from “old” donors with dysplasia** | | | | | |
| **N° donor** | **Age** | **Sex** | **TWIST-1** | **PAR-1** | **MMPs** |
| 37785/10 | 73 | male | **+** | **+** | ND |
| 35772/09 | 76 | male | **+** | **+** | ND |
| 14672/11 | 79 | male | **+** | **+** | ND |
| 14062 | 80 | male | **+** | **+** | ND |
